# Estimating a time-to-event distribution from right-truncated data in an epidemic: A review of methods

**DOI:** 10.1177/09622802211023955

**Published:** 2021-12-21

**Authors:** Shaun R Seaman, Anne Presanis, Christopher Jackson

**Affiliations:** 1 47959 MRC Biostatistics Unit, University of Cambridge, UK

**Keywords:** Coronavirus disease, Cox regression, failure time, identifiability, relative efficiency, right-truncation, survival analysis

## Abstract

Time-to-event data are right-truncated if only individuals who have experienced the event by a certain time can be included in the sample. For example, we may be interested in estimating the distribution of time from onset of disease symptoms to death and only have data on individuals who have died. This may be the case, for example, at the beginning of an epidemic. Right truncation causes the distribution of times to event in the sample to be biased towards shorter times compared to the population distribution, and appropriate statistical methods should be used to account for this bias. This article is a review of such methods, particularly in the context of an infectious disease epidemic, like COVID-19. We consider methods for estimating the marginal time-to-event distribution, and compare their efficiencies. (Non-)identifiability of the distribution is an important issue with right-truncated data, particularly at the beginning of an epidemic, and this is discussed in detail. We also review methods for estimating the effects of covariates on the time to event. An illustration of the application of many of these methods is provided, using data on individuals who had died with coronavirus disease by 5 April 2020.

## Introduction

Data on time to an event are said to be right truncated if they come from a set of individuals who have been randomly sampled from a population using a sampling mechanism that selects only individuals who have experienced the event by a given time, called the truncation time. An example is data on the time from onset of coronavirus disease (COVID-19) symptoms to death collected from a sample of individuals who all developed symptoms and died by 5 April 2020. Among individuals in the population whose symptoms began on, say, 20 March, only those whose time from symptom onset to death was less than 16 days could be included in the sample. For these people, the truncation time is 16 days. Likewise, among those whose onset was on 31 March, only those whose time to death was less than 5 days could be included. Their truncation time is 5 days. Among the sampled individuals, the proportion whose time to death is less than t days (e.g. t=14 days) will be greater than the proportion in the population of people who (eventually) die with COVID-19. So, a naive estimate of the distribution of time to death in this population will be biased. Moreover, the average time to death in sampled individuals whose symptom onset was on 31 March will be shorter than the average time in those whose onset was on 20 March, even if time to death is independent of onset time in the population.

Ideally, we would have a random sample of individuals who experience an initial event (e.g. onset of COVID-19 symptoms) that places them at risk of a final event of interest (e.g. death with COVID-19) and follow them to see how many experience the final event and the times from initial to final event. However, this is not always feasible. For example, in the case of COVID-19 many people will have experienced symptoms but never reported them. Another example is HIV/AIDS, where many people will not have discovered they are infected with HIV (the initial event) until they were diagnosed with AIDS (the final event).

The analysis of right-truncated data requires statistical methods that account for the fact that each of the sampled individuals must have experienced the final event by their truncation time. The purpose of this article is to review such methods in the context of an infectious disease epidemic, like COVID-19.

Much of the statistical methodology for right-truncated data was developed in the 1980s and early 1990s in the context of the HIV/AIDS epidemic (e.g. ^1,2^). However, some of it predated the 1980’s. For example, some methods described in this article involve the idea of reversing the time axis, that is, counting backwards in time from the final event to the intial event. This reversal has the effect of making right-truncated data left-truncated. The Lynden-Bell^3^ estimator is the extension of the original Kaplan-Meier estimator to handle left truncation. Some of the other methods are based on Turnbull’s general algorithm for estimating a distribution function under general patterns of censoring or truncation ^4^. Recently, the emergence of COVID-19 has highlighted the need for methods that handle right truncation. Data from early in the epidemic have been used to estimate the distributions of time from infection to symptom onset, symptom onset to hospitalisation, symptom onset to death, and hospitalisation to death. Although many researchers have accounted for the right truncation of the data (e.g. ^5–7^ some have not done so or it is unclear whether they have (e.g. ^8–10^).

An important issue when estimating the distribution of time to event using right-truncated data is that of identifiability. When the maximum truncation time that can be observed in the sample is less than the maximum time to event in the population, the time-to-event distribution can only be estimated up to a constant of proportionality. In the article, we shall pay particular attention to this issue.

The structure of the article is as follows. In the section ‘Estimating marginal distribution of time to event’ we look at methods that estimate the marginal distribution of time from onset to death in the population of people who (eventually) die from the infection. Some of these methods model both time of onset, and hence truncation time, and time from onset to death, whereas others model only the time from onset to death. Some methods are parametric; others, non-parametric. We review these methods and investigate their asymptotic relative efficiency (ARE) for estimating the mean time from onset to death. Section ‘Estimating and testing covariate effects’ looks at methods for estimating the effect of covariates on the distribution of time to death, and for testing independence between covariates and time to death. These include parametric and semi-parametric methods. An illustration of the application of some of these methods to COVID-19 deaths is described in the section ‘Application to COVID-19’. Section ‘Discussion’ contains some practical recommendations and a brief discussion of more general truncation patterns and of censoring.

## Estimating marginal distribution of time to event

Let X and Y denote the times of an individual’s initial and final events, respectively, with 0≤X≤Y . These two events could be, respectively, onset of COVID-19 symptoms and death from COVID-19. Alternatively, they could be, for example, infection with COVID-19 and hospitalisation, or HIV seroconversion and AIDS diagnosis. Let T=Y−X denote the individual’s time from initial to final event; we call this the ‘delay’. We assume that the support of X includes zero and that X and T are either both continuous or both discrete variables; if they are discrete, we suppose, without loss of generality, that they take integer values and we interpret integrals as sums. Unless stated otherwise, we shall assume T is independent of X . Let $f_{T}^{*}(t)$ and $F_{T}^{*}(t)$ denote, respectively, the probability density (or mass) function of T and the distribution function of T .

We obtain an i.i.d. sample, $\left(x_{1},t_{1}),\ldots ,(x_{n},t_{n}\right)$ , from the probability distribution of (X,T) given X+T≤τ for some τ>0 , that is, from

(1) $$f_{X,T}(x,t|X+T\leq \tau )=\frac{f_{X}(x)f_{T}^{*}(t)\;I(x+t\leq \tau )}{\int_{0}^{\tau }f_{X}(x^{\prime })\;F_{T}^{*}(\tau -x^{\prime })\;dx^{\prime }.}$$

where $f_{X}(x)$ denotes the conditional probability density (or mass) function of X given X≤τ , and I(.) denotes the indicator function. If X and T are discrete, we shall assume, again without loss of generality, that τ is an integer. We should like to estimate $F_{T}^{*}(t)$ , but first we need to discuss whether this is possible.

### Identifiability

The maximum truncation time in the sample is τ . If this is greater than the maximum delay in the population, then $F_{T}^{*}(\tau )=1$ and $F_{T}^{*}(t)$ can be estimated from the data. If, on the other hand, the maximum truncation time is less than the maximum delay in the population, then $F_{T}^{*}(\tau )<1$ and $F_{T}^{*}(t)$ is only (non-parametrically) identified up to a constant of proportionality. This is because the sampling mechanism does not allow us to observe values of T greater than τ , and so the data do not tell us what proportion, $1-F_{T}^{*}(\tau )$ , of individuals in the population have T>τ . This lack of identifiability is manifest in equation (1): if $F_{T}^{*}(\tau )<1$ and the probability density (or mass) function and distribution function of T are instead $f_{T}(t)=f_{T}^{*}(t)/F_{T}^{*}(\tau )$ and $F_{T}(t)=F_{T}^{*}(t)/F_{T}^{*}(\tau )$ ( 0<t≤τ ), then the joint distribution of X and T given X+T≤τ is

(2) $$\frac{f_{X}(x)\;\{f_{T}^{*}(t)/F_{T}^{*}(\tau )\}\;I(x+t\leq \tau )}{\int_{0}^{\tau }f_{X}(x^{\prime })\;\{F_{T}^{*}(\tau -x^{\prime })/F_{T}^{*}(\tau )\}\;dx^{\prime }}$$

which is still equal to the right-hand side of equation (1), because the two $F_{T}^{*}(\tau )$ terms in (2) cancel out.

Unlike $f_{T}^{*}(t)$ and $F_{T}^{*}(t)$ , the functions $f_{T}(t)$ and $F_{T}(t)$ are identifiable from the data $\left(x_{1},t_{1}),\ldots ,(x_{n},t_{n}\right)$ . They are, respectively, the conditional probability density (or mass) function and conditional distribution function of T given T≤τ .

In the absence of other information or assumptions, $F_{T}(t)$ (or equivalently $f_{T}(t)$ ) is all we can estimate. Obviously, if we know from other information that $F_{T}^{*}(\tau )=1$ , then estimating $F_{T}(t)$ is the same as estimating $F_{T}^{*}(t)$ . Likewise, if we know that, for example, $F_{T}^{*}(\tau )=0.8$ , then $F_{T}^{*}(t)=F_{T}(t)\times 0.8$ , and so we can estimate $F_{T}^{*}(t)$ . Alternatively, if we assume a parametric model for $F_{T}^{*}(t)$ and fit this model to the data, then $F_{T}^{*}(\tau )$ (and hence $F_{T}^{*}(t)$ for all t ) will be estimated, because $F_{T}^{*}(\tau )$ is a deterministic function of the model parameters. However, as we shall now illustrate, this estimate of $F_{T}^{*}(\tau )$ relies on extrapolation of the parametric model beyond the range of the data.

### Parametric modelling of joint distribution of X and T

#### A joint-conditional likelihood

We can estimate $F_{T}^{*}(t)$ by parameterising the distributions of X and T in terms of distinct parameters λ and θ , respectively, and maximising the likelihood function corresponding to equation (1), viz.

(3) $$L_{1}=L_{1}(\lambda ,\theta )=\prod_{i=1}^{n}\left\{\;f_{X}(x_{i};\lambda )f_{T}^{*}(t_{i};\theta )/\int_{0}^{\tau }f_{X}(x^{\prime };\lambda )F_{T}^{*}(\tau -x^{\prime };\theta )\;dx^{\prime }\right\}$$

For example, if we assume $f_{X}(x;\lambda )\propto \exp\;(\lambda x)$ and $T∼\text{Gamma}(\theta_{1},\theta_{2})$ , then

$$L_{1}=\frac{1}{{\left(\int_{0}^{\tau }\exp\;(\lambda x^{\prime })\int_{0}^{\tau -x^{\prime }}t^{{}^{\prime }(\theta_{1}-1)}\exp\;(-\theta_{2}t^{\prime })\;dt^{\prime }\;dx^{\prime }\right)}^{n}}\prod_{i=1}^{n}\exp\;(\lambda x_{i})t_{i}^{\theta_{1}-1}\exp\;(-\theta_{2}t_{i})$$

We might call $L_{1}$ the ‘joint-conditional’ likelihood, because it is based on the joint distribution of X and T and is conditional on the final event occurring by time τ .

Notice that equation (3) can be equivalently written as

(4) $$L_{1}=L_{1}(\lambda ,\theta )=\prod_{i=1}^{n}\left\{\;f_{X}(x_{i};\lambda )f_{T}(t_{i};\theta )/\int_{0}^{\tau }f_{X}(x^{\prime };\lambda )F_{T}(\tau -x^{\prime };\theta )\;dx^{\prime }\right\}$$

This highlights that $L_{1}$ depends on the distribution of T only through $F_{T}(t)$ , its conditional distribution given T≤τ . In the example given immediately above, this distribution is

(5) $$F_{T}(t)=\frac{\int_{0}^{t}t^{\prime \left(\theta_{1}-1\right)}\exp\;(-\theta_{2}t^{\prime })\;dt^{\prime }}{\int_{0}^{\tau }t^{\prime \left(\theta_{1}-1\right)}\exp\;(-\theta_{2}t^{\prime })\;dt^{\prime }}\;\;(0<t\leq \tau )$$

Any distribution $F_{T}^{*}(t)$ for which equation (5) describes $F_{T}(t)=F_{T}^{*}(t)/F_{T}^{*}(\tau )$ would yield the same likelihood $L_{1}$ . Thus, the data do not distinguish between $T∼\text{Gamma}(\theta_{1},\theta_{2})$ and, for example, $F_{T}^{*}(t)=F_{T}(t)$ . In the former case, $F^{*}(\tau )=\theta_{2}^{\theta_{1}}\int_{0}^{\tau }t^{{}^{\prime }(\theta_{1}-1)}\exp\;(-\theta_{2}t^{\prime })\;dt^{\prime }/\Gamma (\theta_{1})$ ; in the latter case, $F^{*}(\tau )=1$ . Another possibility is that $F_{T}^{*}(t)=F_{T}(t)\times 0.001$ , in which case $F^{*}(\tau )=0.001$ . In short, $F^{*}(\tau )$ could theoretically be anywhere between 0 and 1 and only $F_{T}(t)$ is non-parametrically identified.

In practice, we might believe that the parametric model accurately describes the whole of the delay distribution, or we might have additional information that makes us confident that $F_{T}^{*}(\tau )=1$ , or the data themselves might suggest that $F_{T}^{*}(\tau )$ equals 1 or is close to 1. The latter might be the case if there were a reasonably large number of sampled individuals with truncation times close to τ and all or almost all of these individuals had delays that were far less than τ . The absence of any longer delays among these people who could have been sampled even if their delays had been longer might lead one to conclude that longer delays are rare. However, caution is warranted, because there remains a possibility that the distribution of delays is bimodal, with a fraction of the population having much longer delays than the rest. For example, this might be the case for COVID-19 if some very seriously ill patients are kept alive on ventilators for a long period of time.

We should be careful not to be misled by a good fit of the parametric model to the data. We can assess the fit on this parametric model only over the range t∈[0,τ] . In all three examples of distributions that give rise to equation (5), this fit is perfect. In particular, if the proportion, $\theta_{2}^{\theta_{1}}\int_{0}^{\tau }t^{\left(\theta_{1}-1\right)}\exp\;(-\theta_{2}t)\;dt/\Gamma (\theta_{1})$ , of the probability mass of the $\text{Gamma}(\theta_{1},\theta_{2})$ distribution that lies in the interval [0,τ] is far from 1, then we are only judging the fit of the model in the early part of the distribution. Note that while it is common in time-to-event studies (without right truncation) for administrative censoring to prevent assessment of the fit of a parametric model in the tail of the distribution, this censoring prevents $F_{T}^{*}(t)$ from being (non-parametrically) identified only for times t after the censoring time.

#### An equivalent likelihood.

So far, we have assumed the data $\left(x_{1},t_{1}),\ldots ,(x_{n},t_{n}\right)$ represent a sample from the population and that the sample size n is fixed. An alternative framework involves assuming that initial events are generated from a (non-homogeneous) Poisson process with rate h(t) at time t , the delays T are independent of the initial event times, and we observe all N individuals for whom X+T≤τ ^2^. Now N is a random variable with N∼Poisson(αC) , where $\alpha =\int_{0}^{\tau }h(t)\;dt$ and $C=C(\lambda ,\theta )=\int_{0}^{\tau }f_{X}(x^{\prime };\lambda )F_{T}(\tau -x^{\prime };\theta )\;dx^{\prime }.$ Also, conditional on N=n , $\left(X_{1},T_{1}),\ldots ,(X_{n},T_{n}\right)$ are i.i.d. with distribution given by equation (1) with $f_{X}(x)=h(x)/\alpha$ . Hence  the joint distribution of $\left(N,X_{1},T_{1},\ldots ,X_{N},T_{N}\right)$ is

$$\begin{array}{rl} \;f_{N,X_{1},T_{1},\ldots ,X_{N},T_{N}}(n,x_{1},t_{1},\ldots ,x_{n},t_{n})= & \frac{\alpha^{n}\exp\;(-\alpha C)}{n!}\\ & \times \prod_{i=1}^{n}f_{X}(x_{i})f_{T}(t_{i})\;I(x_{i}+t_{i}\leq \tau ) \end{array}$$

which gives rise to the likelihood function

(6) $$L_{2}=L_{2}(\alpha ,\lambda ,\theta )=\alpha^{n}\exp\;\{-\alpha C(\lambda ,\theta )\}\prod_{i=1}^{n}f_{X}(x_{i};\lambda )f_{T}(t_{i};\theta )$$

Kalbfleisch and Lawless^2^ show that the maximum likelihood (ML) estimates and observed and estimated Fisher Information for (λ,θ) are the same whether they are obtained from $L_{1}$ or $L_{2}$ . Thus, θ (and λ ) can be estimated from whichever of these likelihoods is most computationally convenient, even if the assumption that the initial events are generated from a Poisson process does not hold (as might be the case, for example, if the initial events occur in clusters).

#### Applications.

Kalbfleisch and Lawless^2^ illustrate the use of $L_{2}$ . They estimate the distribution of time T from infection to onset of AIDS in blood transfusion patients. A common assumption is that at the beginning of an epidemic, cases arise from a Poisson process with rate $h(t)=\lambda_{0}\exp\;(\lambda t)$ . This means that $\alpha =\lambda_{0}\{\exp\;(\lambda \tau )-1\}/\lambda$ and $f_{X}(x;\lambda )=\lambda \exp\;(\lambda x)/\{\exp\;(\lambda \tau )-1\}$ . Kalbfleisch and Lawless make this assumption and assume that T has a Weibull distribution. They obtain the ML estimate of (λ,θ) using a Fisher scoring algorithm. Another example of the use of $L_{2}$ is given by Cox and Medley,^11^ who estimate the distribution of the time T taken for an AIDS diagnosis to be reported to the Communicable Disease Surveillance Centre. They allow the rate of AIDS diagnoses to be increasing sub-exponentially, by using $h(t;\lambda )=\lambda_{0}\exp\;(\lambda_{1}t+\lambda_{2}t^{2})$ , and test the null hypothesis that $\lambda_{2}=0$ . They consider several parametric models for the distribution of the reporting delay T .

Salje et al.^7^ use equation (1), the basis of the likelihood $L_{1}$ , to estimate $f_{T}(t)$ , but do not use ML. In their application, T is the time from hospitalisation with COVID-19 to death. They assume $f_{X}(x)$ , the distribution of hospitalisation times in the population, is known and model $f_{T}^{*}(t)$ as a mixture of an exponential distribution and a log normal distribution. They estimate the parameters of this mixture distribution by finding the values that minimise the sum of squared differences between the distribution of $f_{T}(t|X+T\leq \tau )$ implied by equation (1) and the observed distribution of delays T in the sample.

### Modelling the distribution of T

#### Parametric modelling.

A third likelihood function that can be used to estimate θ comes from factorising $f_{X,T}(x,t|X+T\leq \tau )$ as $f_{X}(x|X+T\leq \tau )\times f_{T}^{*}(t|T\leq \tau -x)$ and using only the second factor. This yields the likelihood

$$L_{3}=L_{3}(\theta )=\prod_{i=1}^{n}\frac{f_{T}^{*}(t_{i};\;\theta )}{F_{T}^{*}(t_{i};\;\theta )}=\prod_{i=1}^{n}\frac{f_{T}(t_{i};\;\theta )}{F_{T}(\tau -x;\;\theta )}$$

For example, if we assume $T∼\text{Gamma}(\theta_{1},\theta_{2})$ , then

(7) $$L_{3}=\prod_{i=1}^{n}\frac{t_{i}^{\theta_{1}-1}\exp\;(-\theta_{2}t_{i})}{\int_{0}^{\tau -x_{i}}t^{\prime \left(\theta_{1}-1\right)}\exp\;(-\theta_{2}t^{\prime })\;dt^{\prime }}.$$

We might refer to $L_{3}$ as the ‘conditional-on-initial (event time)’ likelihood. The issues regarding the identifiability of $F_{T}^{*}(t)$ that were discussed in section ‘Parametric modelling of joint distribution of X and T ’ continue to apply when $L_{3}$ , rather than $L_{1}$ , is used.

#### Non-parametric modelling

$L_{3}$ is also the basis of a non-parametric estimator, ${\hat{F}}_{T}^{\left(\mathrm{NP}\right)}(t)$ , of $F_{T}(t)$ . This estimator is obtained by applying the familiar Kaplan-Meier estimator in reverse-time, that is, to τ−T . By reversing time, right truncation of T (i.e. T must be ≤τ−X ) becomes left truncation of τ−T (i.e. τ−T must be ≥X ). Left truncation (or ‘late entry’) is easily handled by the original Kaplan-Meier estimator (or more accurately, the Lynden-Bell^3^ estimator, which extends the Kaplan-Meier estimator to handle left truncation). For simplicity, we shall now describe the estimator ${\hat{F}}_{T}^{\left(\mathrm{NP}\right)}(t)$ for discrete T ; Lagakos et al.^12^ describe the corresponding estimator for continuous T .

Let $D_{j}=\sum_{i=1}^{n}I(T_{i}=j)$ be the number of delays observed to equal j ( j=0,…,τ ), and let $M_{j}=\sum_{i=1}^{n}I(T_{i}\leq j\leq \tau -X_{i})$ be the number of delays observed to be at most j in those individuals whose truncation time τ−X is such that a delay of j would have been observed. Let ${\hat{G}}_{j}=D_{j}/M_{j}$ , which is a consistent estimator of P(T=j∣T≤j) , the hazard in reverse time (the usual hazard in forward time is P(T=j∣T≥j) ). Since P(T≤t∣T≤τ)=P(T≤t∣T≤t+1)×P(T≤t+1∣T≤t+2)×…×P(T≤τ−1∣T≤τ) ,

(8) $${\hat{F}}_{T}^{\left(\mathrm{NP}\right)}(t)=\prod_{\;j=t\;+\;1}^{\tau }(1-{\hat{G}}_{j})$$

is a consistent estimator of $F_{T}(t)$ . ${\hat{F}}_{T}^{\left(\mathrm{NP}\right)}(t)$ is asymptotically normally distributed and its variance can be consistently estimated using the following adaptation of the Greenwood formula for the variance of the Kaplan-Meier estimator ^12^: $\hat{\text{Var}}\{{\hat{F}}_{T}^{\left(\mathrm{NP}\right)}(t)\}=\{{\hat{F}}_{T}^{\left(\mathrm{NP}\right)}(t){\}}^{2}\sum_{\;j=t+1}^{\tau }D_{j}/\{M_{j}(M_{j}-D_{j})\}.$ 95% confidence limits for ${\hat{F}}_{T}^{\left(\mathrm{NP}\right)}(t)$ can be calculated as $\exp\;(-\exp\;[\hat{K}(t)\pm 1.96\times \surd \hat{\text{Var}}\{\hat{K}(t)\}]),$ where $\hat{K}(t)=\log\;\{-\log\;{\hat{F}}_{T}^{\left(\mathrm{NP}\right)}(t)\}$ and $\hat{\text{Var}}\{\hat{K}(t)\}=\{\log\;{\hat{F}}_{T}^{\left(\mathrm{NP}\right)}(t){\}}^{-2}\sum_{\;j=t\;+\;1}^{\tau }D_{j}/\{M_{j}(M_{j}-D_{j})\}.$ This guarantees that the confidence interval lies within the interval [0,1] .

An alternative way to calculate the same estimator ${\hat{F}}_{T}^{\left(\mathrm{NP}\right)}(t)$ is to define $Y_{x,t}$ (for x=0,…,τ ; t=0,…,τ−x ) as the number of sampled individuals with X=x and T=t and fit the model $Y_{xt}∼\text{Poisson}\{\exp\;(\lambda_{x}+\theta_{t})\}$ . Now ${\hat{F}}_{T}^{\left(\mathrm{NP}\right)}(t)=\sum_{\;j=0}^{t}\exp\;({\hat{\theta }}_{j})/\sum_{\;j=0}^{\tau }\exp\;({\hat{\theta }}_{j})$ , where ${\hat{\theta }}_{j}$ is the ML estimate of $\theta_{j}$ ^1^. Obtaining an estimate of the variance of ${\hat{F}}_{T}^{\left(\mathrm{NP}\right)}(t)$ this way is, however, more difficult.

The non-parametric estimator ${\hat{F}}_{T}^{\left(\mathrm{NP}\right)}(t)$ can be used to check the fit of a parametric model for T . When doing this, it is important to compare ${\hat{F}}_{T}^{\left(\mathrm{NP}\right)}(t)$ with the parametric estimate of $F_{T}(t)$ , rather than of $F_{T}^{*}(t)$ . For example, if we maximise the likelihood $L_{3}$ given by equation (7), we obtain both an estimate of $F_{T}^{*}(t)=P(T\leq t)$ , the distribution function of a gamma distribution, and an estimate of $F_{T}(t)=F_{T}^{*}(t)/F_{T}^{*}(\tau )=P(T\leq t|T\leq \tau )$ , the distribution function of a truncated gamma distribution. ${\hat{F}}_{T}^{\left(\mathrm{NP}\right)}(t)$ should be compared with the latter.

It is important to notice that only the $M_{\tau }$ individuals with X=0 , and hence a truncation time of τ , contribute to the calculation of ${\hat{G}}_{\tau }$ . Therefore, if $M_{\tau }$ is small, ${\hat{G}}_{\tau }$ will have large variance, which causes ${\hat{F}}_{T}^{\left(\mathrm{NP}\right)}(t)$ also to have large variance not just for t=τ but for all values of t . In this case, it is advisable to choose a value $\tau^{*}<\tau$ such that $M_{\tau^{*}}$ is reasonably large and replace the estimator ${\hat{F}}_{T}^{\left(\mathrm{NP}\right)}(t)$ of $F_{T}(t)$ by ${\hat{F}}_{T}^{\left(\mathrm{NP}.\tau^{*}\right)}(t)=\prod_{\;j=t+1}^{\tau^{*}}(1-{\hat{G}}_{j})$ , which is an estimate of $P(T\leq t|T\leq \tau^{*})$ .

#### Relative efficiency of likelihoods $L_{1}$ and $L_{3}$

Unlike $L_{1}$ (or equivalently $L_{2}$ ), $L_{3}$ does not require a model $f_{X}(x;\lambda )$ to be specified for the distribution of the initial event times. This eliminates the risk that such a model may be misspecified. However, it has the disadvantage that some of the information in the data is being discarded, which makes $L_{3}$ less efficient than $L_{1}$ , especially when τ is small. Brookmeyer and Gail^13^ found that the ML estimator of θ based on $L_{3}$ could be considerably less asymptotically efficient that the estimator based on $L_{3}$ when the density of X is known. Jewell^14^ and Kalbfleisch and Lawless^2^ also comment that the loss of efficiency from using $L_{3}$ can be considerable. In section ‘Study of ARE of estimators’ we carry out a more extensive study of relative efficiency, comparing: (i) $L_{1}$ treating the distribution of X as known; (ii) $L_{1}$ treating this distribution as unknown; (iii) $L_{3}$ ; and (iv) the likelihood $L_{4}$ described in section ‘Modelling the distribution of X given Y ’.

A simple example illustrates the information that $L_{1}$ uses but $L_{3}$ does not. Suppose X and T are discrete, with X equal to either 0 or 1 and T equal to either 0 or 1, and τ=1 . We observe 10 individuals with (X,T)=(0,0) , 10 individuals with (X,T)=(0,1) , and no individuals with (X,T)=(1,0) . If we use $L_{3}$ , we would estimate $f_{T}(0)=f_{T}(1)=0.5$ . Now suppose that we know that $f_{X}(0)=f_{X}(1)=0.5$ . Unlike $L_{3}$ , $L_{1}$ uses this information. Since we have only observed individuals with X=0 , it seems likely that there are quite a few individuals with X=1 whom we have not observed. Since we have not observed them, they must all have T=1 . This suggests that $f_{T}(1)>0.5$ .

In section ‘Parametric modelling of joint distribution of X and T ’, we considered the use of $L_{1}$ only with parametric models. If X and T are discrete variables (possibly formed by discretising continuous variables), the distribution of X can instead be modelled non-parametrically. Writing $\lambda =(\lambda_{0},\ldots ,\lambda_{\tau })$ , with $\lambda_{x}=f_{X}(x)$ , $L_{1}$ then becomes

(9) $$L_{1}^{\left(\mathrm{NP}\right)}=\frac{1}{{\left(\sum_{x=0}^{\tau }\lambda_{x}\;F_{T}(\tau -x^{\prime });\;\theta \right)}^{n}}\prod_{i=1}^{n}\lambda_{x_{i}}\;f_{T}\;(t_{i};\theta )$$

Here, $F_{T}(t;\theta )$ can be a non-parametric or parametric model. Kalbfleisch and Lawless^2^ show that the ML estimate of θ obtained from $L_{1}^{\left(\mathrm{NP}\right)}$ is identical to that obtained from $L_{3}$ . This is true whether T is modelled parametrically or non-parametrically and regardless of how finely time (if continuous) is discretised. This shows that when $L_{1}$ is more efficient than $L_{3}$ , this greater efficiency comes from the modelling assumptions $L_{1}$ makes about the marginal distribution of X .

### Modelling the distribution of X given Y

Verity et al.^5^ proposed a fourth likelihood function, which arises from factorising $f_{X,T}(x,t|X+T\leq \tau )$ as $f_{Y}(y|X+T\leq \tau )\times f_{X}(x|Y=y),$ where y=x+t , and using only the second factor. This factor can be written as

(10) $$f_{X}(x|Y=y)=\frac{f_{Y}(y|X=x;\theta )\times f_{X}(x;\lambda )}{\int_{0}^{y}f_{Y}(y|X=x^{\prime };\theta )\times f_{X}(x^{\prime };\lambda )\;dx^{\prime }}$$

They assume that the initial events follow a Poisson process with rate $h(t)=\lambda_{0}\exp\;(\lambda t)$ and that $T∼\text{Gamma}(\theta_{1},\theta_{2})$ . Equation (10) then becomes

$$\begin{array}{rl} \;f_{X}(x|Y=y)= & \frac{\left(y-x{)}^{\theta_{1}-1}\exp\;\{-\theta_{2}(y-x)\}\times \exp\;(\lambda x)\times I(x\leq y\right)}{\int_{0}^{y}(y-x^{\prime }{)}^{\theta_{1}-1}\exp\;\{-\theta_{2}(y-x^{\prime })\}\times \exp\;(\lambda x^{\prime })\;dx^{\prime }}\\ = & \frac{t^{\theta_{1}-1}\exp\;(-\theta_{2}t)\times \exp\;(-\lambda t)\times I(t\leq y)}{\int_{0}^{y}t^{\prime \left(\theta_{1}-1\right)}\exp\;(-\theta_{2}t^{\prime })\times \exp\;(-\lambda t^{\prime })\;dt^{\prime }}\\ = & \frac{t^{\theta_{1}-1}\exp\;\{-(\theta_{2}+\lambda )t\}}{\int_{0}^{y}t^{\prime \left(\theta_{1}-1\right)}\exp\;\{-(\theta_{2}+\lambda )t^{\prime }\}\;dt^{\prime }}, \end{array}$$

which is the density of a truncated gamma distribution with shape $\theta_{1}$ , rate $\theta_{2}+\lambda$ and truncated to [0,y] . This gives the likelihood

(11) $$L_{4}=L_{4}(\lambda ,\theta )=\prod_{i=1}^{n}\frac{t_{i}^{\theta_{1}-1}\exp\;\{-(\theta_{2}+\lambda )t_{i}\}}{\int_{0}^{y_{i}}t^{\prime \left(\theta_{1}-1\right)}\exp\;\{-(\theta_{2}+\lambda )t^{\prime }\}\;dt^{\prime }}$$

We might refer to $L_{4}$ as the ‘conditional-on-final (event time)’ likelihood. It is evident from equation (11) that only $\theta_{1}$ and $\theta_{2}+\lambda$ are identified. Practical use of $L_{4}$ therefore requires that λ be known.

Verity et al.^5^ analysing data on 24 COVID-19 deaths that occurred in China very early in the epidemic, assumed λ=0.14 per day and estimated that the mean time from onset of symptoms to death was 19 (95% CI 16–50) days. They did not explain why they used $L_{4}$ , rather than $L_{1}$ . In view of the small sample, it was probably impractical to estimate both λ and θ . However, $L_{1}$ could have been used instead, also with λ fixed at 0.14. The appeal of $L_{4}$ may have been ease of use: it is just the likelihood of a truncated gamma distribution. Another advantage of $L_{4}$ relative to $L_{1}$ , which may have been relevant, is that the former is a valid likelihood no matter how individuals are sampled, provided that the sampling probabilities depend only on Y . Validity of $L_{1}$ as a likelihood requires a simple random sample of individuals with X+T≤τ .

Equation (11) is derived from the assumptions that $f_{X}(x)\propto \exp\;(\lambda t)$ and T has a gamma distribution. The ‘conditional-on-final’ likelihood could also be derived from other assumed distributions for X and T , but would not have the form of a truncated gamma distribution.

### Study of ARE of estimators

We carried out a study of the AREs of the ML estimators of the expected delay, E(T) , based on (i) $L_{1}$ with known λ (‘ $L_{1}^{\mathrm{kwn}}$ ’), (ii) $L_{3}$ , and (iii) $L_{4}$ (with known λ ), all relative to the ML estimator based on (iv) $L_{1}$ with unknown λ (‘ $L_{1}^{\mathrm{est}}$ ’). We assumed $f_{x}(x)\propto \exp\;(\lambda x)$ and $T∼\text{Gamma}(\theta_{1},\theta_{2})$ , and considered multiple scenarios defined by different combinations of values of λ , $\theta_{1}$ , $\theta_{2}$ and τ . For the distribution of X , we used λ=0 , 0.035, 0.07, 0.14 and 0.28. For the distribution of T , we used $\theta_{1}=1$ , 2, 5 and 10, and set $\theta_{2}=\theta_{1}/19$ , so that T has mean E(T)=19 . The mean of 19 (days) was chosen because it was the estimate calculated by Verity et al. (2020) early in the COVID-19 pandemic. τ was varied from 10 to 60 . Note that the AREs are invariant to the choice of E(T)=19 , in the sense that they do not change if λ and $\theta_{2}$ are both multiplied by some constant and τ is divided by the same constant (keeping $\theta_{1}$ unchanged).

To calculate an ARE, we first calculated the asymptotic variance of each of the four ML estimators of $\theta =(\theta_{1},\theta_{2})$ . Then we obtained the corresponding asymptotic variance of each of the four ML estimators of E(T) using the Delta Method. The ratio of the asymptotic variances of two estimators of E(T) is their ARE. The formulae used for these calculations are given in the Supplemental Materials.

**Figure 1.**
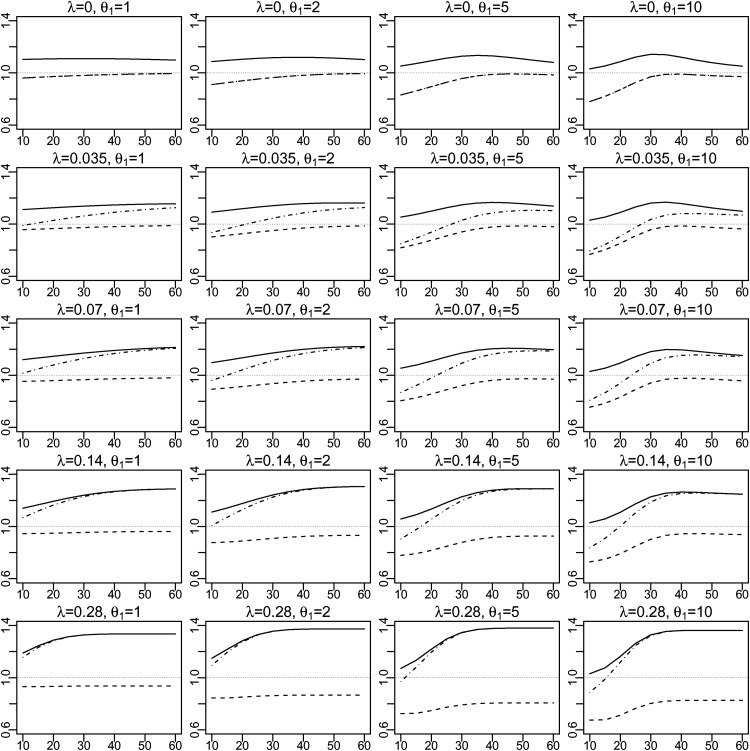
For each of five values of λ and of four values of $\theta_{1}$ , graph shows the asymptotic relative efficiency (ARE) of the estimator of E(T) based on (i) $L_{1}^{\mathrm{kwn}}$ (solid line), (ii) $L_{3}$ (broken line) and (iii) $L_{4}$ (dot-dash line) all compared to (iv) $L_{1}^{\mathrm{est}}$ . The x -axis of each graph is τ and the y -axis is the ARE.

Figure 1 shows the results. Each row corresponds to a different value of λ ; each column, to a different value of $\theta_{1}$ . The x -axis of each graph represents τ and the y -axis represents the ARE. The ARE of $L_{1}^{\mathrm{kwn}}$ (relative to $L_{1}^{\mathrm{est}}$ ) varies from slightly over 1.0 to about 1.4. It increases with λ ; it also increases with τ , at least when τ≤30 . The ARE of $L_{3}$ (relative to $L_{1}^{\mathrm{est}}$ ) varies from 0.67 to almost 1. It decreases with increasing λ or $\theta_{1}$ , and mostly increases with τ . In particular, it is close to 1 when X is uniformly distributed ( λ=0) and T is exponentially distributed ( $\theta_{1}=1$ ), and is 0.67 when λ=0.28 , $\theta_{1}=10$ and τ=10 . When X is uniformly distributed ( λ=0 ), $L_{4}$ has exactly the same efficiency as $L_{3}$ (see Supplemental Material for proof). However, as λ increases, $L_{4}$ becomes relatively more efficient, and approaches the efficiency of $L_{1}^{\mathrm{kwn}}$ , especially for larger values of τ .

We also calculated the AREs for the ML estimators of the median of T . These were almost identical to the AREs for the expectation, E(T) (see Supplemental Material).

## Estimating and testing covariate effects

Let Z be a covariate or vector of covariates. We assume, unless stated otherwise, that Z is independent of X . We may be interested in testing whether T is independent of Z and/or estimating the effect of Z on T .

### Parametric models

A parametric model $f_{T}^{*}(t|Z=z;\beta )$ can be specified for the distribution of T given Z . For example, T might be assumed to have a gamma distribution with $\log\;E(T|Z)=\beta_{0}+\beta_{1}Z$ and shape parameter $\beta_{2}$ . Then $\beta =(\beta_{0},\beta_{1},\beta_{2})$ can be estimated by maximising $L_{1}$ , $L_{2}$ or $L_{3}$ . Just as in the case with no covariates (section ‘An equivalent likelihood’), the ML estimate and Fisher information for β obtained from $L_{1}$ and $L_{2}$ are identical. ^2^If Z is a function of X , this same method can still be applied using either $L_{1}$ or $L_{3}$ . A likelihood ratio test or Wald test can be used for the null hypothesis that one or more elements of the vector β equal zero.

Kalbfleisch and Lawless^2^ give an example of using $L_{2}$ with a Weibull regression model for the effect of age Z on time T from HIV infection to AIDS diagnosis.

### Semi-parametric models

Brookmeyer and Liao^15^ propose a generalisation of the discrete-time estimator ${\hat{F}}_{T}^{\left(\mathrm{NP}\right)}(t)$ to estimate covariate effects. Fit the τ binomial regression models $g\{P(T=j|T\leq j\leq \tau -X,Z=z)=\beta_{0j}+\beta_{1}z$ ( j=1,…,τ ) simultaneously, where g is a specified link function. Let ${\hat{\beta }}_{0j}$ and ${\hat{\beta }}_{1}$ denote the resulting estimates. Then calculate ${\hat{F}}_{T}(t|Z=z)=\prod_{\;j=t+1}^{\tau }\{1-g^{-1}({\hat{\beta }}_{0j}+{\hat{\beta }}_{1}z)\}$ . In the absence of covariates, this is equivalent to ${\hat{F}}_{T}^{\left(\mathrm{NP}\right)}(t)$ . Brookmeyer and Liao recommend using the complementary log link, g(p)=log{−log(1−p)} , because the model then implies $F_{T}(t|Z=z)=\{F_{T}(t|Z=0){\}}^{\exp\;(\beta_{1}z)}$ , which provides an interpretation of $\beta_{1}$ . The null hypothesis that $\beta_{1}$ (or a subvector of a vector $\beta_{1}$ ) equals zero can be tested using a likelihood ratio test or Wald test. This method can also be used when Z is a function of X .

Kalbfleisch and Lawless^16^ extend the method of Brookmeyer and Liao to continuous time and derive score tests of the null hypothesis that $\beta_{1}=0$ . Following a similar approach, Lagakos et al.^12^ had earlier described a log rank test for testing independence of T and a binary covariate Z .

The Poisson regression approach to calculating ${\hat{F}}_{T}^{\left(\mathrm{NP}\right)}(t)$ , described in section ‘Non-parametric modelling’, is extended by Brookmeyer and Damiano^1^ to perform a likelihood ratio test of the global null hypothesis that $\beta_{1}=0$ . This is done by including interaction terms in the Poisson model. This approach is less useful, however, for testing whether a set of covariates is conditionally independent of T given another set of covariates or for estimating covariate effects. ^15^

### Proportional hazards models

A common assumption in both parametric and semi-parametric models for a time-to-event outcome is that hazards are proportional. Brookmeyer and Liao’s^15^ semi-parametric model, described in section ‘Semi-parametric models’, assumes proportional hazards, but in reverse time. This differs from the usual proportional hazards assumption, which is in forward time and states that hazard ratios $\beta_{1}$ are constant over time and $1-F_{T}^{*}(t|Z=z)=\{1-F_{T}^{*}(t|Z=0){\}}^{\exp\;(\beta_{1}z)}$ . Brookmeyer and Liao’s model implies that when $\beta_{1}z>0$ (respectively, $\beta_{1}z<0$ ), the (forward-time) hazard ratio comparing Z=z to Z=0 is initially greater (less) than one and decreases (increases) monotonically over time, becoming equal to one at time τ−1 .

Finkelstein et al.^17^ show that the (forward-time) proportional hazards assumption suffices to identify $F_{T}^{*}(\tau )$ , provided that the hazard ratios of the covariates in the model do not all equal zero. When the hazard ratios all equal zero, $F_{T}^{*}(\tau )$ is not identified, just as in the non-parametric case with no covariates. Finkelstein et al. describe how to fit the semi-parametric proportional hazards model by ML. Provided that the hazard ratios of the covariates do not all equal zero, this provides estimates of the hazard ratios and $F_{T}^{*}(\tau )$ . Unfortunately, the identification of $F_{T}^{*}(\tau )$ relies entirely on the proportional hazard assumption. If this does not hold, the estimate of $F_{T}^{*}(\tau )$ can be heavily biased. Moreover, if there is only one covariate Z in the model, its hazard ratio is estimated very imprecisely. Finkelstein et al. discourage the use of their method for estimating $F_{T}^{*}(\tau )$ or the hazard ratio of a single covariate. When there are multiple covariates with non-zero hazard ratios in the model (and possibly additional covariates with zero hazard ratio), Finkelstein et al. find that these hazard ratios can be estimated more precisely. However, it is unclear how big might be the effect of a small violation of the proportional hazards assumption on the bias of these estimates when $F_{T}(\tau )<1$ . Alioum and Commenges^18^ suggest that when there is only one covariate, its hazard ratio could be estimated twice, once with $F_{T}^{*}(\tau )$ fixed at its lowest value considered plausible, and once at its highest plausible value. However, the resulting range of hazard ratios in their example is very wide.

Perhaps the main use of Finkelstein et al.’s method is for hypothesis testing with multiple covariates. Brookmeyer and Liao (1990), Lagakos et al. (1988) and Brookmeyer and Damiano (1989) described simpler hypothesis testing methods. However, if one wants to test whether one set of covariates is independent of T given another set of covariate, then Lagakos et al.’s log rank test cannot be used, and although the binomial regression model of Brookmeyer and Liao or the Poisson model of Brookmeyer and Damiano could be used, the parameters in these models do not have interpretations as standard hazard ratios, whereas those in Finkelstein et al.’s model do.

Finkelstein et al.’s method involves estimating the baseline hazard. Vakulenko-Lagun et al.^19^ propose a simpler Cox regression approach with inverse probability weighting. This uses a modification of the Cox partial likelihood, which does not depend on the baseline hazard. This method involves weighting the observed individuals so that they represent both themselves and those individuals who were not observed because of the right truncation. The method requires either that $F_{T}^{*}(\tau )=1$ or that $F_{T}^{*}(\tau |Z=0)$ is known. If $F_{T}^{*}(\tau |Z=0)$ is unknown, a sensitivity analysis can be performed, analysing the data using a range of plausible values of $F_{T}^{*}(\tau |Z=0)$ . This method can be applied using the R package *coxrt*. We are not aware of software being available for implementing Finkelstein et al.’s method.

## Application to COVID-19

The first case of COVID-19 in the UK was reported on 30 January 2020. Public Health England (PHE) receives reports every day of COVID deaths from National Health Service England, the Demographics Batch Service (DBS) and Health Protection Teams (HPT). DBS and HPT also report date of symptom onset, when available, for these deceased individuals. Here we illustrate some of the methods discussed above using data available early in the epidemic, specifically at 9 April. We estimate the distribution of time (‘delay’) from symptom onset to death and investigate the effects of sex and age on this distribution. This is intended only as a simple illustration of methods; results should be interpreted with caution.

**Figure 2.**
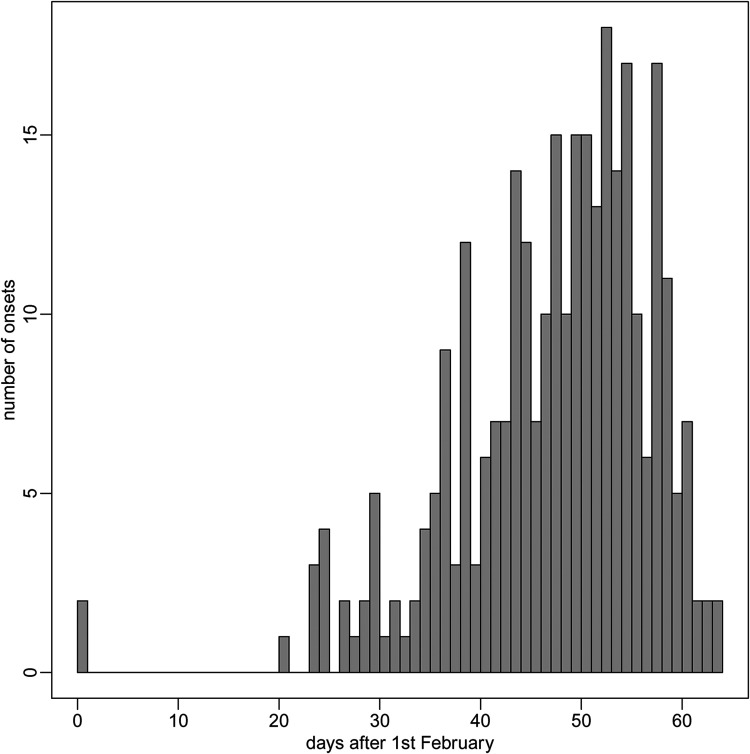
Distribution of symptom onset times in the sample of 304 individuals.

To allow for reporting delays, we exclude deaths occurring between 6 and 9 April; around 80% of deaths are reported within 4 days. ^20^Of the remaining 7415 deaths, the symptom onset date was known for 316 (4.3%). Of these 316, we excluded 12 because of missing sex or age. The remaining 304 constitute the sample we shall use. 180 were male and 124 female; 25 were aged under 65, 33 aged 65–74, 100 aged 75–84, and 146 were aged over 85. Figure 2 shows the distribution of onset times X . The distribution is skewed, with most onsets being in the second half of March. This reflects exponential growth in the early phase of the epidemic. The earliest observed onset date was 1 February (time zero); the two individuals with onset on that day have the maximum truncation time of τ=64 days. Only 13 other individuals have onsets before 2 March (time 30), and so truncation times τ−X greater than 34 days; most truncation times are less than 20 days.

**Figure 3.**
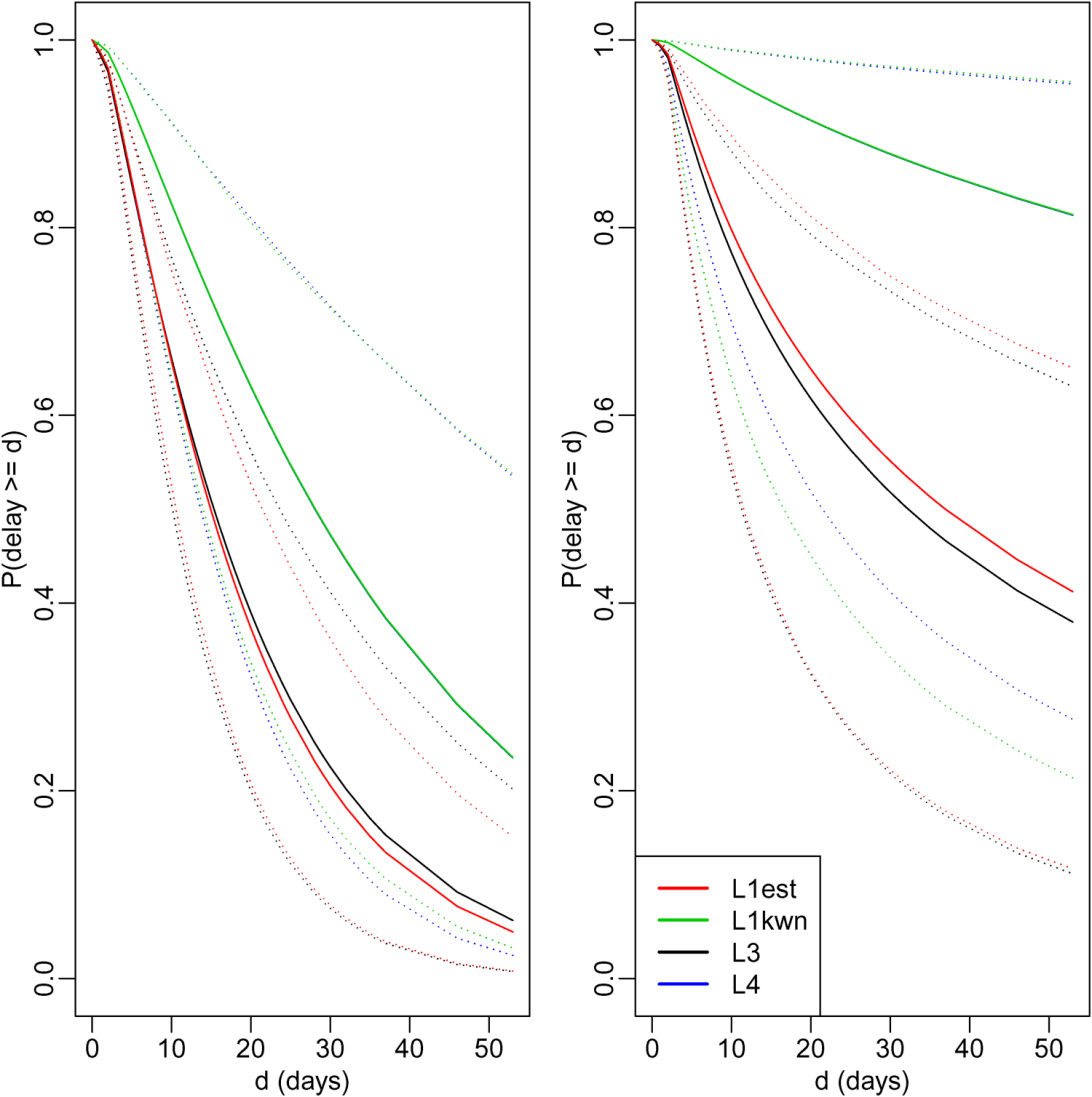
Estimated survival curves from the gamma model (left) and log normal model (right), obtained using likelihoods $L_{1}^{\mathrm{est}}$ , $L_{1}^{\mathrm{kwn}}$ , $L_{3}$ and $L_{4}$ . Dotted lines represent 95% confidence intervals (Estimates using $L_{1}^{\mathrm{kwn}}$ and $L_{4}$ are so close they may be hard to distinguish.).

The mean delay in the sample is 7.1 days (range: 0–52 days). As only those who die by 5 April can be included in the sample (right truncation), the mean in the population could be much greater. Using the R package *flexsurv*, ^21^ we estimated the distribution of delays in the population by fitting two parametric models: a gamma distribution and a log normal distribution. Each was fitted in four ways: by maximising $L_{1}$ with unknown λ (‘ $L_{1}^{\mathrm{est}}$ ’), $L_{1}$ with known λ (‘ $L_{1}^{\mathrm{kwn}}$ ’), $L_{3}$ and $L_{4}$ . For $L_{1}^{\mathrm{kwn}}$ and $L_{4}$ , we assumed λ=0.14 , the estimate calculated early in the epidemic by Verity et al.^5^. Figure 3 shows the estimates of the survival distribution (i.e. $1-F_{T}^{*}(t)$ ). These are quite diverse. For example, estimated survival at 30 days varies from 0.21 to 0.88. However, as expected, these estimates are all greater than the proportion (0.02) of the sample who have delays greater than 30 days. The estimates from the log normal model are systematically greater than those from the gamma model, and all estimates have wide associated confidence intervals. For a given model, the estimates from $L_{3}$ and $L_{1}^{\mathrm{est}}$ are similar to one another, and those from $L_{1}^{\mathrm{kwn}}$ and $L_{4}$ are almost identical to one another. This is perhaps not surprising, given our findings in section ‘Study of ARE of estimators’ for the gamma distribution. There we found that: (1) when λ≥0.07 , $L_{3}$ and $L_{1}^{\mathrm{est}}$ have similar asymptotic efficiency, unless τ is small compared to the mean delay E(T) ; and (2) $L_{3}$ and $L_{1}^{\mathrm{est}}$ have similar asymptotic efficiencies when λ<0.14 and the shape parameter of the gamma distribution equals 1. For the COVID data, the estimates of E(T) from the gamma model varied from 19 for $L_{3}$ or $L_{1}^{\mathrm{est}}$ to 36 for $L_{1}^{\mathrm{kwn}}$ or $L_{4}$ , and the estimates of the shape varied from 1.16 to 1.24.

For both the gamma and log normal models, the estimates of λ from $L_{1}^{\mathrm{est}}$ were 0.11 (95% CI 0.09–0.12). The difference between this estimate and the assumed value of λ=0.14 used by $L_{1}^{\mathrm{kwn}}$ and $L_{4}$ may explain why the estimates of survival from $L_{1}^{\mathrm{est}}$ and $L_{3}$ are lower than those from $L_{1}^{\mathrm{kwn}}$ and $L_{4}$ . Compared to λ=0.11 , λ=0.14 implies a later average onset time, E(X) , in the population, and hence a higher proportion of people in that population that have delays too long to be sampled ( T>τ−X ). This implied greater extent of right truncation implies a greater difference between the mean delay in the sample and the mean delay in the population.

**Figure 4.**
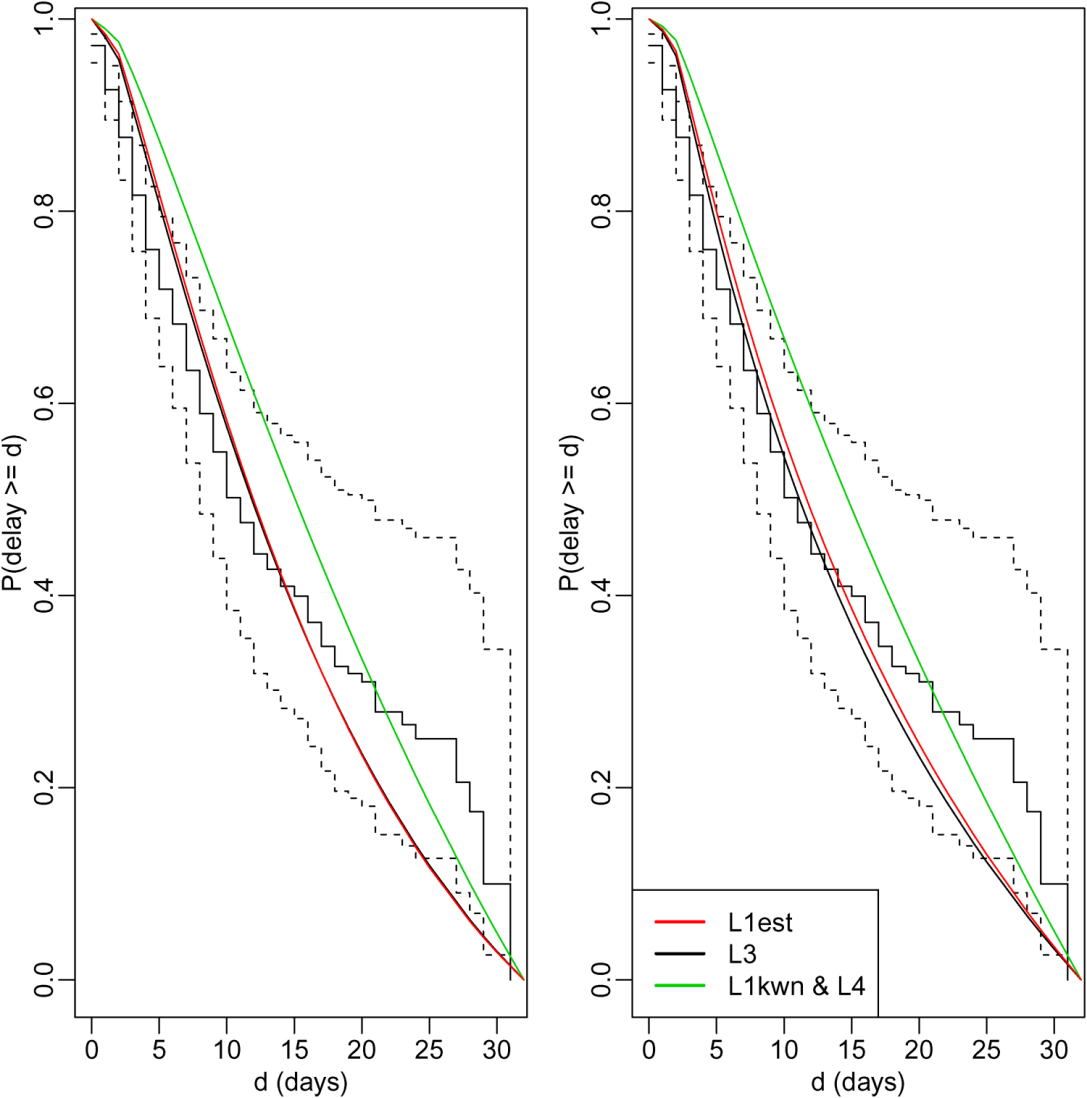
Comparison of non-parametric estimate (step function) of survival conditional on delay being less than 31 days with corresponding estimates from the gamma model (left) and log normal model (right). Dotted lines represent 95% confidence intervals for the non-parametric estimate. Estimates using $L_{1}^{\mathrm{kwn}}$ and $L_{4}$ are so close that they are shown by a single line, and estimates using $L_{1}^{\mathrm{est}}$ and $L_{3}$ are so close that they may be difficult to distinguish.

We used the non-parametric estimate of survival conditional on $T\leq \tau^{*}$ , that is, $1-{\hat{F}}_{T}^{\left(\mathrm{NP}\right)}(t)$ , to assess the fit of the parametric models. To avoid having wide confidence intervals for ${\hat{F}}_{T}^{\left(\mathrm{NP}\right)}(t)$ , we used $\tau^{*}=31$ . This ensures that at least $M_{\tau^{*}}=20$ individuals had truncation times of $\tau^{*}$ days. Figure 4 compares $1-{\hat{F}}_{T}^{\left(\mathrm{NP}\right)}(t)$ with the corresponding parametric estimates of survival conditional on $T\leq \tau^{*}$ , that is, with the estimates of $1-F_{T}(t)/F_{T}(\tau^{*})$ . The fit of the models using $L_{1}^{\mathrm{est}}$ and $L_{3}$ is reasonable during the first 15 days, but less good thereafter. The fit when $L_{1}^{\mathrm{kwn}}$ or $L_{4}$ is used is considerably worse, presumably because the data do not support the choice of λ=0.14 . Note that the difference between the *conditional* (on $T\leq \tau^{*}$ ) survivor curves estimated from the gamma model and the corresponding estimates from the log normal model is much less obvious than the differences between the corresponding *unconditional* survivor functions (shown in Figure 3). This illustrates the point made in sections ‘Identifiability’ and ‘A joint-conditional likelihood’ that two models can give similar estimates of $F_{T}(t)$ and yet very different estimates of $F_{T}^{*}(t)$ .

Next we fitted the gamma and log normal models with sex and age group (0–64, 65–74, 75–84 and ≥85 ) as covariates, again using the R package *flexsurv*. This was done using the likelihood $L_{3}$ ; *flexsurv* does not currently allow $L_{1}$ or $L_{4}$ to be used with covariates. The gamma (respectively, log normal) model assumes that the log rate (respectively, mean of the log delay) is a linear function of the covariates. Delays were estimated to be longer for males than females and for younger than for older people. Both effects were borderline-significant in the gamma model. Neither was significant in the log normal model, although age was found to be significant when a trend test was used (see Supplemental Materials).

If the gamma or log normal model is misspecified, tests of covariate effects may not be valid. Brookmeyer and Liao’s method allow tests that do not depend on parametric assumptions. Using this method, we found again that delays are longer for males and younger people. Neither effect reached statistical significance, although age was significant when a trend test was used (see Supplemental Materials).

**Figure 5.**
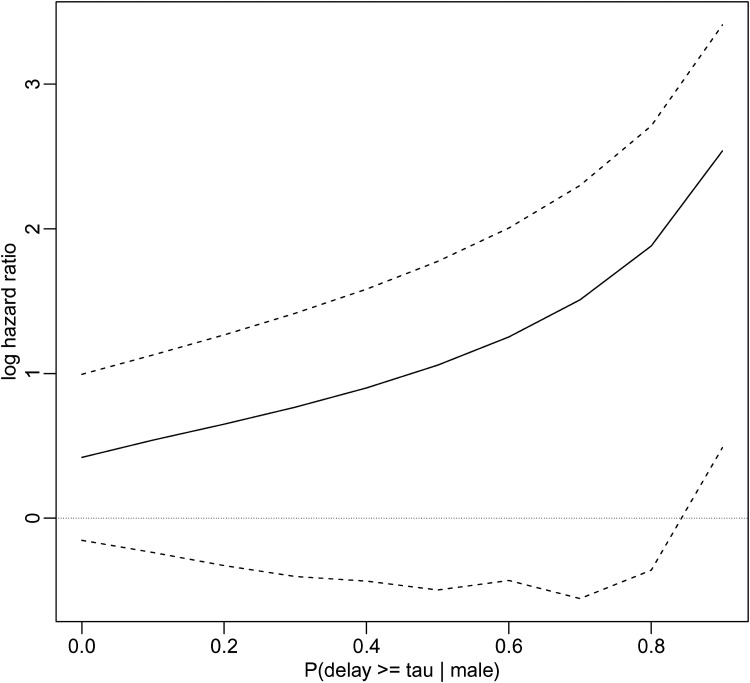
Estimate of log hazard ratio associated with sex=female as a function of P(T≥τ∣sex=male) . Dotted lines indicate 95% confidence limits calculated by bootstrap; these may be unreliable when P(T≥τ∣sex=male) is large (see text).

Finally, we used Vakulenko-Lagun’s et al.’s Cox regression method. This requires that either $F_{T}^{*}(\tau )=1$ or we specify a value for $F_{T}^{*}(t|Z=0)$ . Figure 3 suggests it is unlikely that $F_{T}^{*}(\tau )$ is close to 1. Using the R package *coxrt*, we fitted the model that included the covariate sex, varying $F_{T}^{*}(t|Z=0)$ over the full range from 1.0 to 0.1, where here Z=0 means male. The method uses inverse probability weighting to account for the right truncation, with the weights being functions of a Kaplan-Meier estimate of the truncation time distribution. As explained in the Supplemental Materials, this Kaplan-Meier estimate could not be calculated for our data set, until we excluded the 12 individuals with onset times before 1 March. This makes 1 March the new time zero, and so τ now equals 36. Figure 5 shows how the estimated log hazard ratio associated with being female changes as $P(T\geq \tau |Z=0)=1-F_{T}^{*}(\tau |Z=0)$ changes. Females are estimated to have a greater hazard than males (and hence shorter mean delay) and the estimated log hazard ratio increases as P(T≥τ∣Z=0) increases. However, the confidence intervals, calculated using 1000 bootstrap samples, indicate that this effect is not significant, at least not until P(T≥τ∣Z=0)=0.1 . There were convergence problems: the percentage of bootstrap samples for which convergence was not achieved was 0.0% when P(T≥τ∣Z=0) is 0.2 or less, 0.3% when it is 0.3, 1.4% when it is 0.5, 3.1% when it is 0.7, and 7.9% when it is 0.9. This may make the estimated confidence intervals unreliable for the largest values of P(T≥τ∣Z=0) . We also tried to fit the model with age group, both as a four-level unordered categorical variable and an ordinal categorical variable with linear effect, but the fitting algorithm did not converge. We could, however, fit the model with age as a binary variable, although again with some convergence problems in the bootstrap samples (see Supplemental Materials).

## Discussion

We have considered ML estimation of the marginal distribution of the delay T , using four likelihoods. Likelihoods $L_{1}$ and $L_{2}$ are based on the joint distribution of T and the time of the initial event X ; $L_{3}$ on the distribution of T given X ; and $L_{4}$ on the distribution of T given the time of the final event Y=X+T . Estimates from $L_{1}$ and $L_{2}$ are identical. $L_{3}$ has the advantage of not requiring a model for $f_{X}(x)$ but the disadvantage of yielding the least efficient estimates. $L_{4}$ requires $f_{X}(x)$ to be known exactly. When $f_{X}(x)$ is known, $L_{1}$ is more efficient than $L_{4}$ . $L_{1}$ also has the advantage over $L_{4}$ that it can be used when $f_{X}(x)$ is a function of unknown parameters. However, $L_{4}$ has the advantage that, unlike $L_{1}$ and $L_{3}$ , it yields valid estimates even when the sampling probabilities depend on the actual values of Y , rather than only on whether Y≤τ .

In our study of asymptotic efficiency, we found the ARE of $L_{3}$ relative to $L_{1}$ varied between 0.67 and 1 when $f_{X}(x)$ was unknown. Because $L_{3}$ does not use information on $f_{X}(x)$ , these AREs became more marked when $f_{X}(x)$ was known, varying between 0.58 and 0.92. AREs of 0.67 and 0.58 correspond to reductions in sample size of 33% and 42%, respectively. These AREs were calculated assuming a gamma distribution for the delay and exponential growth over time in the number of initial events. In the early phase of an epidemic, the assumption of exponential growth may be tenable, but it is unlikely to hold later on. More research on the AREs when X and T have other distributions is warranted, as well as on finite-sample relative efficiencies.

The non-parametric estimator, ${\hat{F}}_{T}^{\left(\mathrm{NP}\right)}(t)$ , of the delay distribution has the attraction of not requiring distributional assumptions. It does, however, only estimate the distribution of T conditional on T≤τ ; the unconditional distribution of T is estimable only by using parametric assumptions. One use of ${\hat{F}}_{T}^{\left(\mathrm{NP}\right)}(t)$ is to assess the fit of parametric models over the range t∈[0,τ] . However, the confidence intervals associated with ${\hat{F}}_{T}^{\left(\mathrm{NP}\right)}(t)$ may be very wide at the beginning of an epidemic, when the numbers of sampled individuals with large truncation times τ−X may be small.

To estimate the effects of covariates on the delay, and to test whether these effects are non-zero, $L_{1}$ or $L_{3}$ can be used with parametric models. Brookmeyer and Liao’s (1990) semi-parametric model can also be used, particularly for the purpose of testing whether the effect of a single covariate is zero. The semi-parametric Cox regression method of Vakulenko-Lagun et al. (2019) allows hazard ratios of covariates to be estimated under a standard proportional hazards assumption. However, it does assume that the covariates are independent of the truncation time τ−X , and hence of X . Moreover, it requires that $F_{T}^{*}(\tau )$ be equal to one or that an interval can be specified within which $F_{T}^{*}(\tau )$ is believed to lie. As this interval becomes wider, the uncertainty in the hazard ratios increases. We also had some convergence problems when fitting these models to the COVID-19 data (section ‘Application to COVID-19’).

The R package *flexsurv* can be used to fit parametric models using $L_{1}$ , $L_{3}$ and $L_{4}$ , and also to calculate ${\hat{F}}_{T}^{\left(\mathrm{NP}\right)}(t)$ . Brookmeyer and Liao’s (1990) method can be applied using any software for fitting generalised linear models. The R package *coxrt* applies the method of Vakulenko-Lagun et al. (2019). We have focussed on ML estimation, but also Bayesian analyses can be carried out using the likelihoods $L_{1}$ , $L_{2}$ , $L_{3}$ and $L_{4}$ . Indeed, the analysis of Verity et al. (2020) was Bayesian, using $L_{4}$ with informative priors.

In addition to being right-truncated, Y may be censored. This is easily handled in parametric models by replacing $f_{T}^{*}(t_{i})$ in $L_{1}$ and $L_{3}$ by $F_{T}^{*}(t_{i}^{U})-F_{T}^{*}(t_{i}^{L})$ , where $\left[t_{i}^{L},t_{i}^{U}\right]$ is the interval within which individual i ’s delay is known to lie. If individual i is left-censored, $t_{i}^{L}=0$ ; if right-censored, $t_{i}^{U}=\infty$ . The estimator ${\hat{F}}_{T}^{\left(\mathrm{NP}\right)}(t)$ is easily extended to allow left censoring of Y . ^22^The non-parametric estimator of $F_{T}(t)$ under general censoring of both X and Y and right truncation of Y is described by Sun.^23^Alioum and Commenges (1996) generalise Finkelstein et al.’s (1993) method to allow interval censoring of Y . Double truncation (i.e. simultaneous left and right truncation) of Y is addressed by, among others.^4,24,25,18,23,26,27^The R package *DTDA* ^28^ can be used to calculate the non-parametric estimator of $F_{T}(t)$ when Y is double truncated (it can also be used when Y is only right-truncated, but calculating ${\hat{F}}_{T}^{\left(\mathrm{NP}\right)}(t)$ using equation (8) is faster). Brookmeyer and Gail^13^ showed that when Y is double-truncated and the distributions of X and T are modelled parametrically, the efficiency gain from using $L_{1}$ rather than $L_{3}$ to estimate $f_{T}(t)$ could be considerably greater than the efficiency gains we showed in section ‘Study of ARE of estimators’. In the Supplemental Materials, we extend our study of ARE to double-truncated data and replicate Brookmeyer and Gail’s finding.

$F_{T}(t)$ and $F_{T}^{*}(t)$ describe the distribution of T in the population of individuals who will (eventually) experience the final event. They do not describe what proportion of the population will never experience the final event. An alternative sampling mechanism randomly samples individuals who experience any one of a number of mutually exclusive types of final event by time τ . For example, one might have a random sample from the population of individuals who develop COVID-19 symptoms and go on to die or recover by time τ . This situation of competing risks and right truncation is discussed by Hudgens et al.^29^ and de Una-Alvarez,^30^ who describe how to estimate cumulative incidence functions. These functions describe what proportion of individuals who die or recover by time τ will die by time t and what proportion will recover by time t ( t≤τ ).

In addition to the problems of right-truncation, censoring and competing risks, other issues can complicate the estimation of a time-to-event distribution early in an epidemic. Overton et al.^31^ describes some of these, which include the possibility that some individuals who experience the final event leave the country before being detected, changes over time in the definition of the final event or in the format of the data being collected, and delays in reporting the final event.

## Supplemental Material

sj-pdf-1-smm-10.1177_09622802211023955 - Supplemental material for Estimating a time-to-event distribution from right-truncated data in an epidemic: A review of methods Supplemental material, sj-pdf-1-smm-10.1177_09622802211023955 for Estimating a time-to-event distribution from right-truncated data in an epidemic: A review of methods by Shaun R Seaman, Anne Presanis and Christopher Jackson in Statistical Methods in Medical Research
