# Supplementary material for: Estimating a time-to-event distribution from right-truncated data in an epidemic: A review of methods
Source: Stat Methods Med Res. 2021 Dec 21;31(9):1641–55. doi: 10.1177/09622802211023955 (PMC9465556; doi:10.1177/09622802211023955)
Supplement: sj-pdf-1-smm-10.1177_09622802211023955 - Supplemental material for Estimating a time-to-event distribution from right-truncated data in an epidemic: A review of methods [file sj-pdf-1-smm-10.1177_09622802211023955.pdf]

Supplemental Material for "Estimating a Time-to-Event Distribution from Right-Truncated Data in an Epidemic: a Review of Methods"

By Shaun R. Seaman, Anne Presanis and Christopher Jackson

MRC Biostatistics Unit, Institute of Public Health, Forvie Site, Robinson Way, Cambridge, CB2 0SR.
Corresponding author: Shaun Seaman
shaun.seaman@mrc-bsu.cam.ac.uk

A Formulae for asymptotic variances of maximum likelihood estimators

Here we provide the formulae used in the calculation of the asymptotic relative efficiencies (AREs) reported in Section 2.5. We assume that $f_X(x) \propto \exp(\lambda x)$ for $0 \leq x \leq \tau$. This means that $f_X(x) = \lambda \exp(\lambda x)/\{\exp(\lambda\tau) - 1\}$ if $\lambda > 0$, and $f_X(x) = 1/\tau$ if $\lambda = 0$. We also assume that $T \sim \text{Gamma}(\theta_1, \theta_2)$.

For ease of reading, we shall write θ_1 as α and write θ_2 as β. So, we have

$$f_T(t) = \frac{\beta^\alpha}{\Gamma(\alpha)} t^{\alpha-1} \exp(-\beta t) \qquad (t > 0).$$

We begin by deriving formulae for the Fisher information for L_3.

$$L_3 = L_3(x,t) = \frac{f_T(t)}{F_T(\tau - x)} = \frac{t^{\alpha-1}\exp(-\beta t)}{\int_0^{\tau-x} s^{\alpha-1}\exp(-\beta s)\, ds}.$$

Define

$$A(a,b,c) = \int_0^a (\log s)^b\, s^c \exp(-\beta s)\, ds.$$

Using the fact that

$$\frac{d}{du}(s^u) = \frac{d}{du}[\exp\{\log(s^u)\}] = \frac{d}{du}\{\exp(u\log s)\} = (\log s)\exp(u \log s) = s^u \log s$$

and the quotient rule for differentiation, it can be shown that $\frac{\partial^2}{\partial\alpha^2}\log L_3(x,t)$ is a function only of x and equals

$$\frac{\partial^2}{\partial\alpha^2}\log L_3(x) = \frac{\{A(\tau-x,1,\alpha-1)\}^2 - A(\tau-x,2,\alpha-1)\, A(\tau-x,0,\alpha-1)}{\{A(\tau-x,0,\alpha-1)\}^2} \quad (1)$$

$$\frac{\partial^2}{\partial\beta^2}\log L_3(x) = \frac{\{A(\tau-x,0,\alpha)\}^2 - A(\tau-x,0,\alpha+1)\, A(\tau-x,0,\alpha-1)}{\{A(\tau-x,0,\alpha-1)\}^2} \quad (2)$$

$$\frac{\partial^2}{\partial\alpha\partial\beta}\log L_3(x) = \frac{A(\tau-x,1,\alpha)\, A(\tau-x,0,\alpha-1) - A(\tau-x,0,\alpha)\, A(\tau-x,1,\alpha-1)}{\{A(\tau-x,0,\alpha-1)\}^2}. \quad (3)$$

Finally,

$$
\begin{aligned}
E\left\{\frac{\partial^2}{\partial\alpha^2}\log L_3(X)\right\} &= \int_0^\tau f_X(x \mid X+T \le \tau)\,\frac{\partial^2}{\partial\alpha^2}\log L_3(x)\;dx & (4)\\
E\left\{\frac{\partial^2}{\partial\beta^2}\log L_3(X)\right\} &= \int_0^\tau f_X(x \mid X+T \le \tau)\,\frac{\partial^2}{\partial\beta^2}\log L_3(x)\;dx & (5)\\
E\left\{\frac{\partial^2}{\partial\alpha\partial\beta}\log L_3(X)\right\} &= \int_0^\tau f_X(x \mid X+T \le \tau)\,\frac{\partial^2}{\partial\alpha\partial\beta}\log L_3(x)\;dx & (6)
\end{aligned}
$$

where

$$
\begin{aligned}
f_X(x \mid X+T \le \tau) &= \frac{f_X(x)F_T(\tau-x)}{\int_0^\tau f_X(s)F_T(\tau-s)\;ds}\\
&= \frac{\exp(\lambda x)\;A(\tau-x,0,\alpha-1)}{\int_0^\tau \exp(\lambda s)\;A(\tau-s,0,\alpha-1)\;ds}.
\end{aligned}
$$

Now we derive formulae for the Fisher information for L_1.

$$
L_1 = L_1(x,t) = \frac{\exp(\lambda x)t^{\alpha-1}\exp(\beta t)}{\int_0^\tau \exp(\lambda u)\int_0^{\tau-u} s^{\alpha-1}\exp(-\beta s)\;ds\;du}.
$$

Define

$$
B(a,b,c) = \int_0^\tau u^a\exp(\lambda u)\;A(\tau-u,b,c)\;du.
$$

It can be shown that $\frac{\partial^2}{\partial\lambda^2}\log L_1(x,t)$ depends on neither x nor t, and equals

$$
\begin{aligned}
\frac{\partial^2}{\partial\lambda^2}\log L_1 &= \frac{\{B(1,0,\alpha-1)\}^2 - B(2,0,\alpha-1)\;B(0,0,\alpha-1)}{\{B(0,0,\alpha-1)\}^2} & (7)\\
\frac{\partial^2}{\partial\alpha^2}\log L_1 &= \frac{\{B(0,1,\alpha-1)\}^2 - B(0,2,\alpha-1)\;B(0,0,\alpha-1)}{\{B(0,0,\alpha-1)\}^2} & (8)\\
\frac{\partial^2}{\partial\beta^2}\log L_1 &= \frac{\{B(0,0,\alpha)\}^2 - B(0,0,\alpha+1)\;B(0,0,\alpha-1)}{\{B(0,0,\alpha-1)\}^2} & (9)\\
\frac{\partial^2}{\partial\lambda\partial\alpha}\log L_1 &= \frac{B(0,1,\alpha-1)B(1,0,\alpha-1) - B(1,1,\alpha-1)\;B(0,0,\alpha-1)}{\{B(0,0,\alpha-1)\}^2} & (10)\\
\frac{\partial^2}{\partial\lambda\partial\beta}\log L_1 &= \frac{-B(0,0,\alpha)\;B(1,0,\alpha-1) + B(1,0,\alpha)\;B(0,0,\alpha-1)}{\{B(0,0,\alpha-1)\}^2} & (11)\\
\frac{\partial^2}{\partial\alpha\partial\beta}\log L_1 &= \frac{-B(0,0,\alpha)\;B(0,1,\alpha-1) + B(0,1,\alpha)\;B(0,0,\alpha-1)}{\{B(0,0,\alpha-1)\}^2}. & (12)
\end{aligned}
$$

Now we derive formulae for the Fisher information for L_4.

$$
L_4 = L_4(x,t) = \frac{t^{\alpha-1}\exp\{-(\lambda+\beta)t\}}{\int_0^{x+t} s^{\alpha-1}\exp\{-(\lambda+\beta)s\}\;ds}.
$$

Define

$$
C(a,b,c) = \int_0^a (\log s)^b\;s^c\exp\{-(\lambda+\beta)s\}\;ds.
$$

It can be shown that $\frac{\partial^2}{\partial\alpha^2} \log L_4(x,y)$ is a function only of y and equals

$$
\begin{aligned}
\frac{\partial^2}{\partial\alpha^2} \log L_4(y) &= \frac{\{C(y,1,\alpha-1)\}^2 - C(y,2,\alpha-1)\, C(y,0,\alpha-1)}{\{C(y,0,\alpha-1)\}^2} \\
\frac{\partial^2}{\partial\beta^2} \log L_4(y) &= \frac{\{C(y,0,\alpha)\}^2 - C(y,0,\alpha+1)\, C(y,0,\alpha-1)}{\{C(y,0,\alpha-1)\}^2} \\
\frac{\partial^2}{\partial\alpha\partial\beta} \log L_4(y) &= \frac{C(y,1,\alpha)\, C(y,0,\alpha-1) - C(y,0,\alpha)\, C(y,1,\alpha-1)}{\{C(y,0,\alpha-1)\}^2}.
\end{aligned}
$$

We have

$$
\begin{aligned}
E\left\{\frac{\partial^2}{\partial\alpha^2} \log L_4(Y)\right\} &= \int_0^\tau f_Y(y \mid Y \le \tau)\, \frac{\partial^2}{\partial\alpha^2} \log L_4(y)\, dy && (13) \\
E\left\{\frac{\partial^2}{\partial\beta^2} \log L_4(Y)\right\} &= \int_0^\tau f_Y(y \mid Y \le \tau)\, \frac{\partial^2}{\partial\beta^2} \log L_4(y)\, dy && (14) \\
E\left\{\frac{\partial^2}{\partial\alpha\partial\beta} \log L_4(Y)\right\} &= \int_0^\tau f_Y(y \mid Y \le \tau)\, \frac{\partial^2}{\partial\alpha\partial\beta} \log L_4(y)\, dy && (15)
\end{aligned}
$$

where

$$
\begin{aligned}
f_Y(y \mid Y \le \tau) &\propto f_Y(y) \\
&= \int_0^y f_X(x)\, f_T(y-x)\, dx \\
&\propto \int_0^y \exp(\lambda x)(y-x)^{\alpha-1} \exp\{-\beta(y-x)\}\, dx \\
&\propto \exp(\lambda y) \int_0^y s^{\alpha-1} \exp\{-(\lambda+\beta)s\}\, ds.
\end{aligned}
$$

Hence,

$$
f_Y(y \mid Y \le \tau) = \frac{\exp(\lambda y)\, \psi(y;\alpha,\lambda+\beta)}{\int_0^\tau \exp(\lambda s)\, \psi(s;\alpha,\lambda+\beta)\, ds},
$$

where $\psi(y;\alpha,\lambda+\beta)$ denotes the cumulative distribution function of a gamma distribution with shape α and rate $\lambda+\beta$ evaluated at y.

The functions $A(.)$, $B(.)$ and $C(.)$ and the expected second derivatives in equations (4)–(6) and (13)–(15) can all be calculated using numerical integration.

Calculation of AREs of ML estimators of mean and median of T

Having calculated the asymptotic variance matrix of an ML estimator of (α,β) from the Fisher information for either (λ,α,β) or (α,β), as appropriate, the next step is to use the Delta Method to get the corresponding asymptotic variance of the ML estimator of $E(T) = \alpha/\beta$ and the asymptotic variance of the ML estimator of the median of T. The Delta method requires derivatives for $E(T)$ and the median of T with respect to α and β. For the median of T we used the approximation for the median of a Gamma distribution given by Berg and Pedersen (2006)[1]. The ratio of the asymptotic variances of two estimators of $E(T)$ or of the median of T is their ARE.

As Figure A1 shows, the AREs of the ML estimators of the median of T were very similar to corresponding AREs of the ML estimators of $E(T)$.

Special case: $\lambda = 0$

When $\lambda = 0$, the Fisher information matrices of L_3 and L_4 are identical, and so the ML estimators of α, β and any functions (e.g. $E(T)$ and the median of T) of α and β based on L_3 have identical asymptotic efficiencies to the ML estimators based on L_4. This is because, when $\lambda = 0$, we have $f_Y(y \mid Y \leq \tau) = f_X(\tau - y \mid X + T \leq \tau)$, $L_4(x,t) = L_3(\tau - x - t, t)$ and $C(a,b,c) = A(a,b,c)$. Consequently,

$$\begin{aligned} E\left\{\frac{\partial^2}{\partial\alpha^2}\log L_4(Y)\right\} &= \int_0^\tau f_Y(y \mid Y \leq \tau)\,\frac{\partial^2}{\partial\alpha^2}\log L_4(y)\,dy \\ &= \int_0^\tau f_X(\tau - y \mid X + T \leq \tau)\,\frac{\partial^2}{\partial\alpha^2}\log L_3(\tau - y)\,dy \\ &= \int_0^\tau f_X(s \mid X + T \leq \tau)\,\frac{\partial^2}{\partial\alpha^2}\log L_3(s)\,ds \\ &= E\left\{\frac{\partial^2}{\partial\alpha^2}\log L_3(X)\right\}. \end{aligned}$$

and, similarly,

$$\begin{aligned} E\left\{\frac{\partial^2}{\partial\beta^2}\log L_4(Y)\right\} &= E\left\{\frac{\partial^2}{\partial\beta^2}\log L_3(X)\right\} \\ \text{and}\quad E\left\{\frac{\partial^2}{\partial\alpha\partial\beta}\log L_4(Y)\right\} &= E\left\{\frac{\partial^2}{\partial\alpha\partial\beta}\log L_3(X)\right\}. \end{aligned}$$

B Relative efficiency with double truncation

In this section, we report the results of a study of ARE when the final event times Y are not only right-truncated at time τ but also left-truncated at time ω, i.e. individuals are randomly sampled conditional on $\omega \leq Y \leq \tau$. We first describe the necessary modifications to the formulae given above when Y is both left- and right-truncated.

B.1 Modifications to formulae

We begin with L_3. Now the appropriate likelihood L_3 is

$$L_3 = \frac{f_T(t)}{F_T(\tau - x) - F_T(\omega - x)} = \frac{t^{\alpha-1}\exp(-\beta t)}{\int_{\max(\omega-x,0)}^{\tau-x} s^{\alpha-1}\exp(-\beta s)\,ds}.$$

So, we need to replace all the terms $A(\tau - x, b, c)$ in equations (1)–(3) by $A^*(\omega - x, \tau - x, b, c)$, where

$$A^*(a_1, a_2, b, c) = \int_{\max(a_1,0)}^{a_2} (\log s)^b\, s^c \exp(-\beta s)\,ds.$$

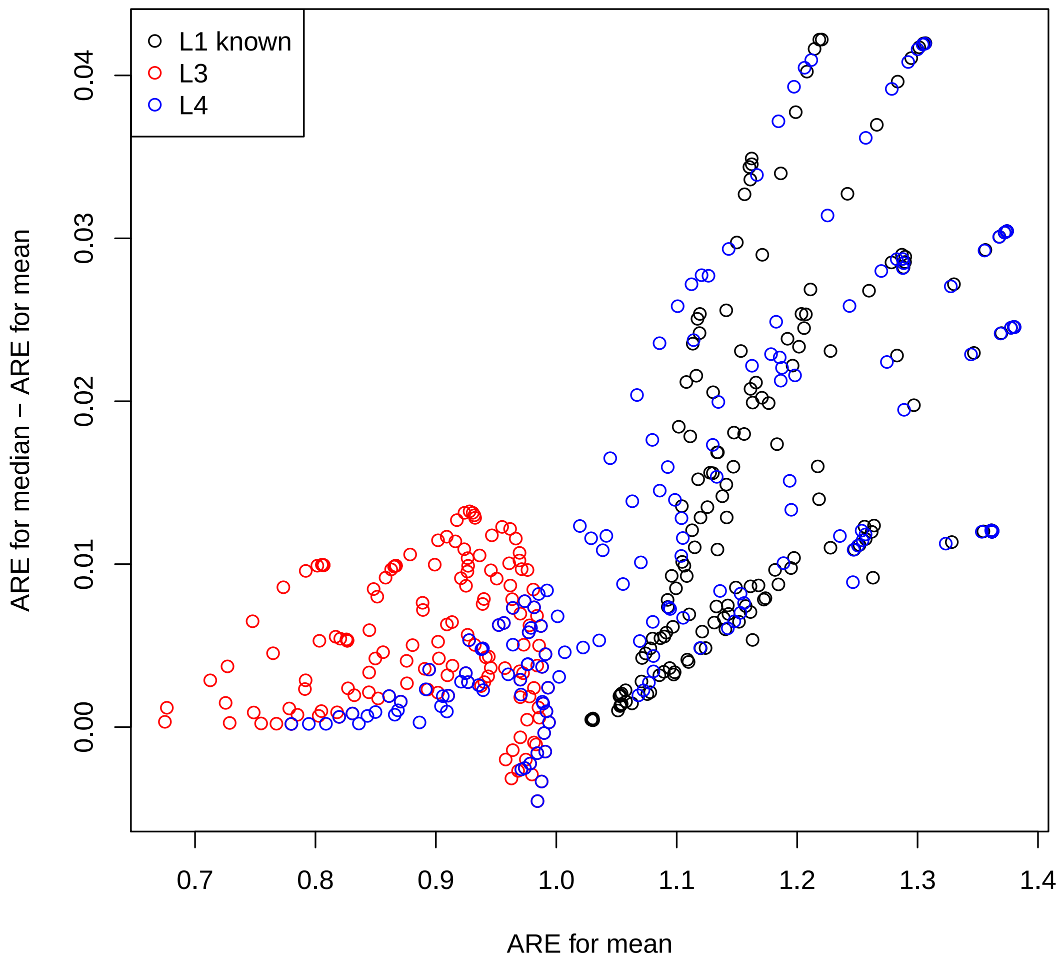

Figure A1: Comparison of AREs of the ML estimators of the median of T with the AREs of the ML estimators of $E(T)$. The ARE of a ML estimator of $E(T)$ is plotted on the x-axis, and the difference between this ARE and the ARE of the corresponding ML estimator of the median of T is plotted on the y-axis. The ARE of the estimator based on L_1 with known λ is shown by a black dot, the ARE of the estimator based on L_3 is shown by a red dot, and the ARE of the estimator based on L_4 is shown by a blue dot. All these AREs are relative to the estimator based on L_1 with unknown λ. Only AREs for scenarios in which the shape of the gamma distribution is greater than one are shown, since the AREs for the mean and median are exactly equal when the shape equals one.

Also, we replace $f_X(x \mid X+T \leq \tau)$ in equations (4)–(6) by

$$f_X(x \mid \omega \leq X+T \leq \tau) = \frac{\exp(\lambda x)\ A^*(\omega - x, \tau - x, 0, \alpha - 1)}{\int_0^\tau \exp(\lambda s)\ A^*(\omega - s, \tau - s, 0, \alpha - 1)\ ds}.$$

Now consider L_1. We need to replace all the terms $B(a,b,c)$ in equation (7)–(12) by $B^*(a,b,c)$, where

$$B^*(a,b,c) = \int_0^\tau u^a \exp(\lambda u)\ A^*(\omega - u, \tau - u, b, c)\ du.$$

Finally, consider L_4. In equations (13)–(15) we replace $f_Y(y \mid Y \leq \tau)$ by $f_Y(y \mid \omega \leq Y \leq \tau)$, which is given by

$$f_Y(y \mid \omega \leq Y \leq \tau) = \frac{\exp(\lambda y)\ \psi(y; \alpha, \lambda + \beta)}{\int_\omega^\tau \exp(\lambda s)\ \psi(s; \alpha, \lambda + \beta)\ ds}$$

if $\omega \leq y \leq \tau$ and which equals zero otherwise.

B.2 Results

Figure 1 in Section 2.5 showed the AREs of the ML estimators of $E(T)$ based on i) L_1 with known λ (black line), ii) L_3 (red line) and iii) L_4 (blue line) all compared to iv) L_1 with unknown λ. Those are the AREs when the final event time Y is right-truncated at time τ.

Figure A2 shows the corresponding AREs when Y is not only right-truncated at time τ but is also left-truncated at time $\omega = 10$. Figures A3 and A4 show the corresponding results when the left truncation time is, respectively, $\omega = 20$ and $\omega = 30$.

For ease of viewing, Figures A5–A7 are the same as Figures A2–A4, but with the y-axis restricted to the range $[0.6, 1.0]$.

Comparison with Brookmeyer and Gail (1988)'s result

Brookmeyer and Gail (1988)[2] considered the case of $\lambda = 0$ (uniformly distributed X), $\theta_1 = 1$ (exponentially distributed T), right truncation at time $\tau = 7E(T)/4$ and left truncation at time $\omega = 9E(T)/8$. When, as it is true of our data-generating mechanism, $E(T) = 19$, these truncation times are $\tau = 7 \times 19/4 = 33.25$ and $\omega = 9 \times 19/8 = 21.38$. Brookmeyer and Gail calculated that the ARE of L_3 with *known* θ_1 compared to L_1 with known λ *and known* θ_1 is 0.16. The top-left graph in Figure A3 approximately represents this case, although the AREs in this graph are calculated treating θ_1 as *unknown.* This graph shows that when $\omega = 20$ and $\tau = 33$ the ARE of L_3 compared to L_1 with unknown λ is 0.76 and the ARE of L_1 with unknown λ compared to L_1 with unknown λ is 2.73. Hence, the ARE of L_3 compared to L_1 with known λ is $0.76/2.73 = 0.28$, which is greater than Brookmeyer and Gail's 0.16.

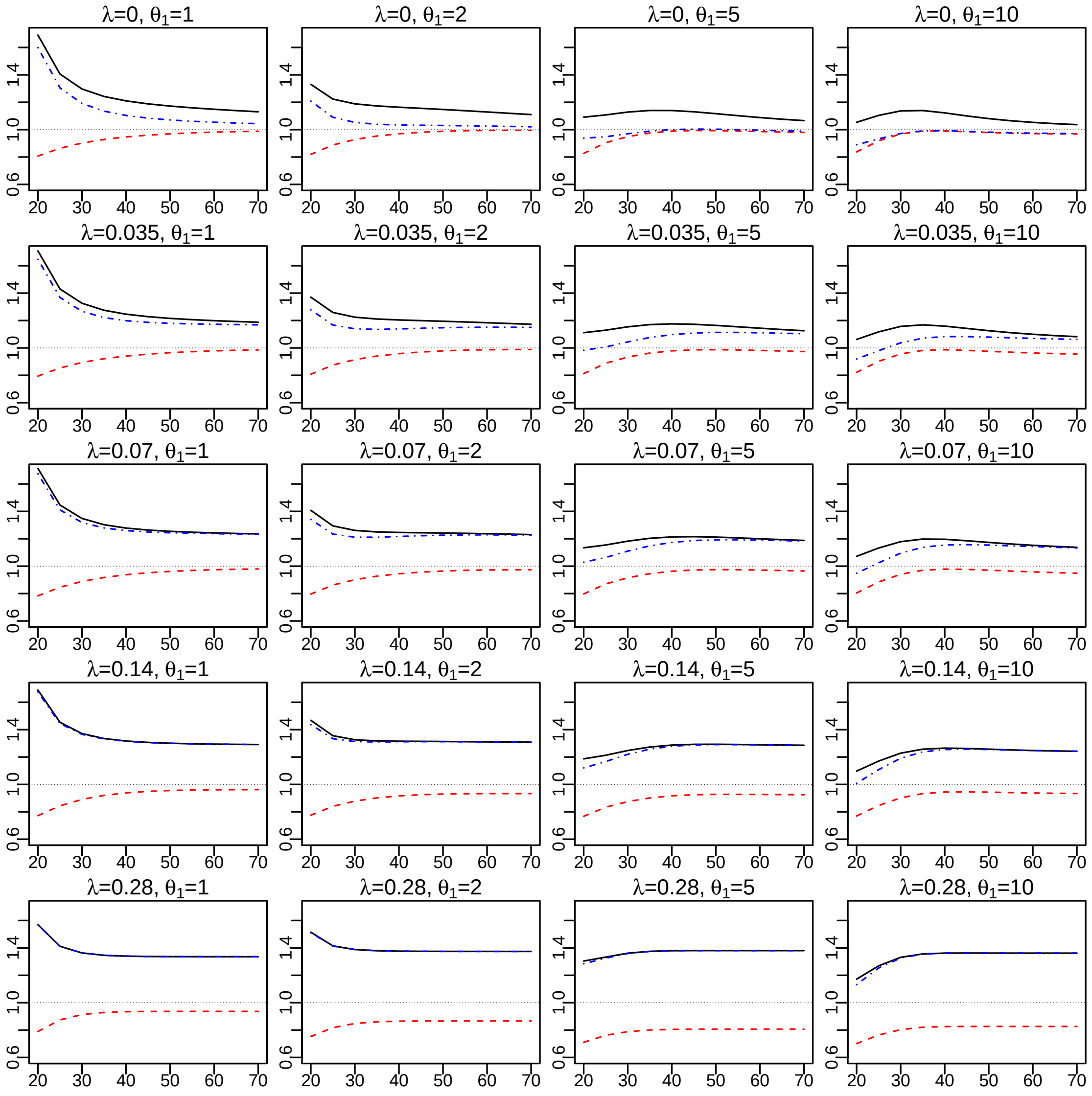

Figure A2: Asymptotic relative efficiency (ARE) of the estimator of $E(T)$ based on i) L_1 with known λ (black line), ii) L_3 (red line) and iii) L_4 (blue line) all compared to iv) L_1 with unknown λ. The x-axis of each graph is τ and the y-axis is the ARE. The final event times Y are double truncated within the interval $[10, \tau]$.

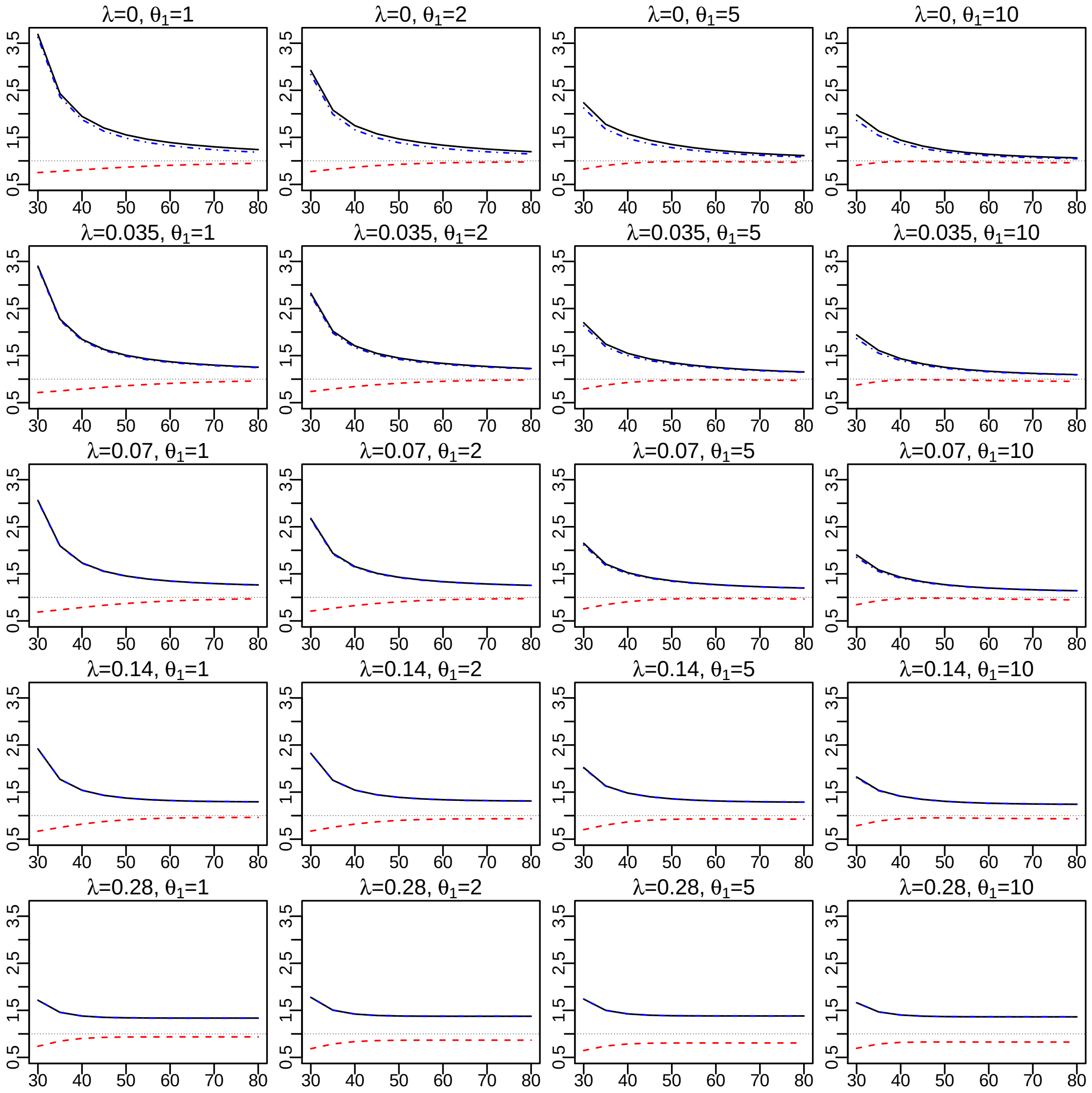

Figure A3: Asymptotic relative efficiency (ARE) of the estimator of $E(T)$ based on i) L_1 with known λ (black line), ii) L_3 (red line) and iii) L_4 (blue line) all compared to iv) L_1 with unknown λ. The x-axis of each graph is τ and the y-axis is the ARE. The final event times Y are double truncated within the interval $[20, \tau]$.

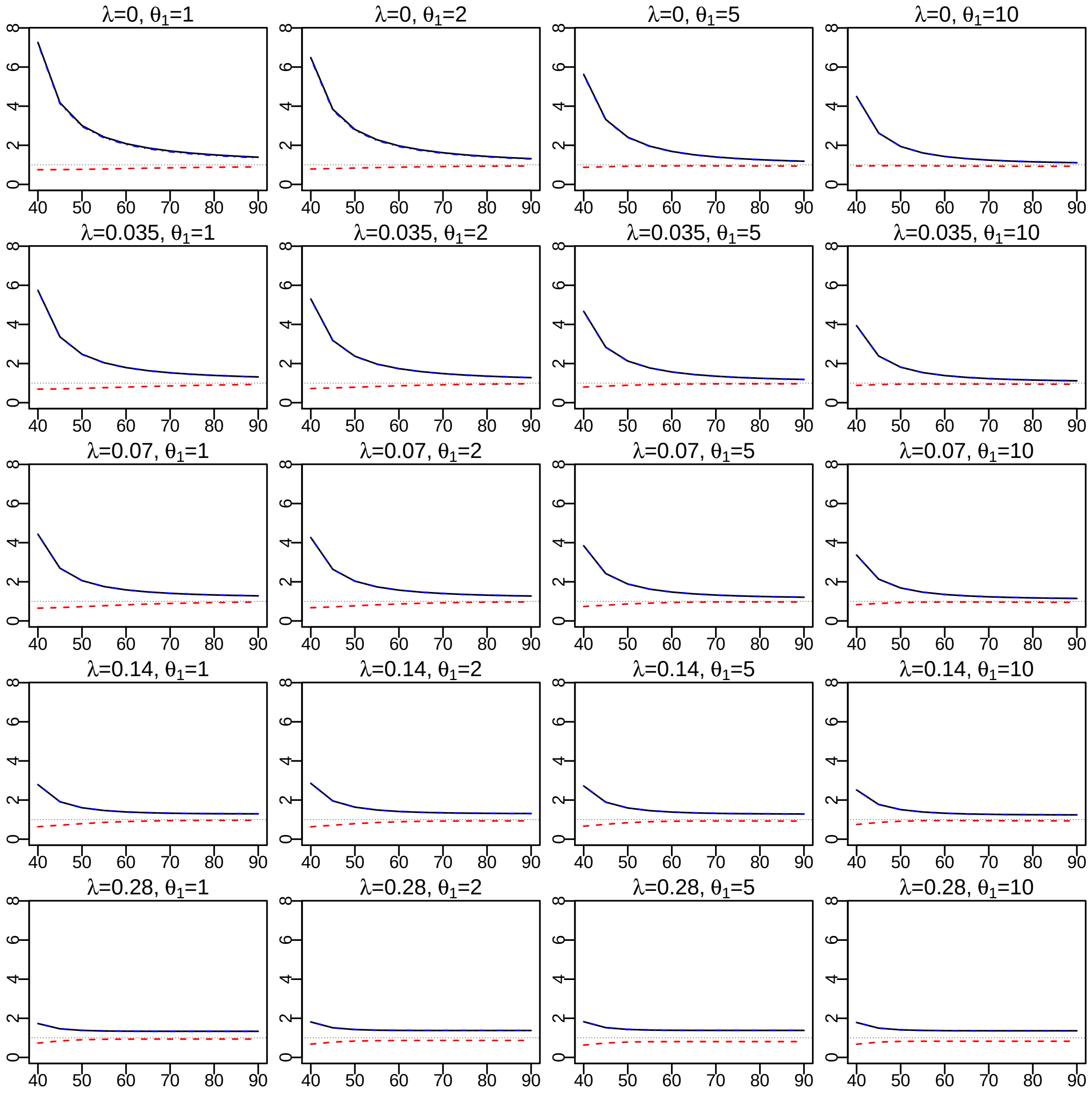

Figure A4: Asymptotic relative efficiency (ARE) of the estimator of $E(T)$ based on i) L_1 with known λ (black line), ii) L_3 (red line) and iii) L_4 (blue line) all compared to iv) L_1 with unknown λ. The x-axis of each graph is τ and the y-axis is the ARE. The final event times Y are double truncated within the interval $[30, \tau]$.

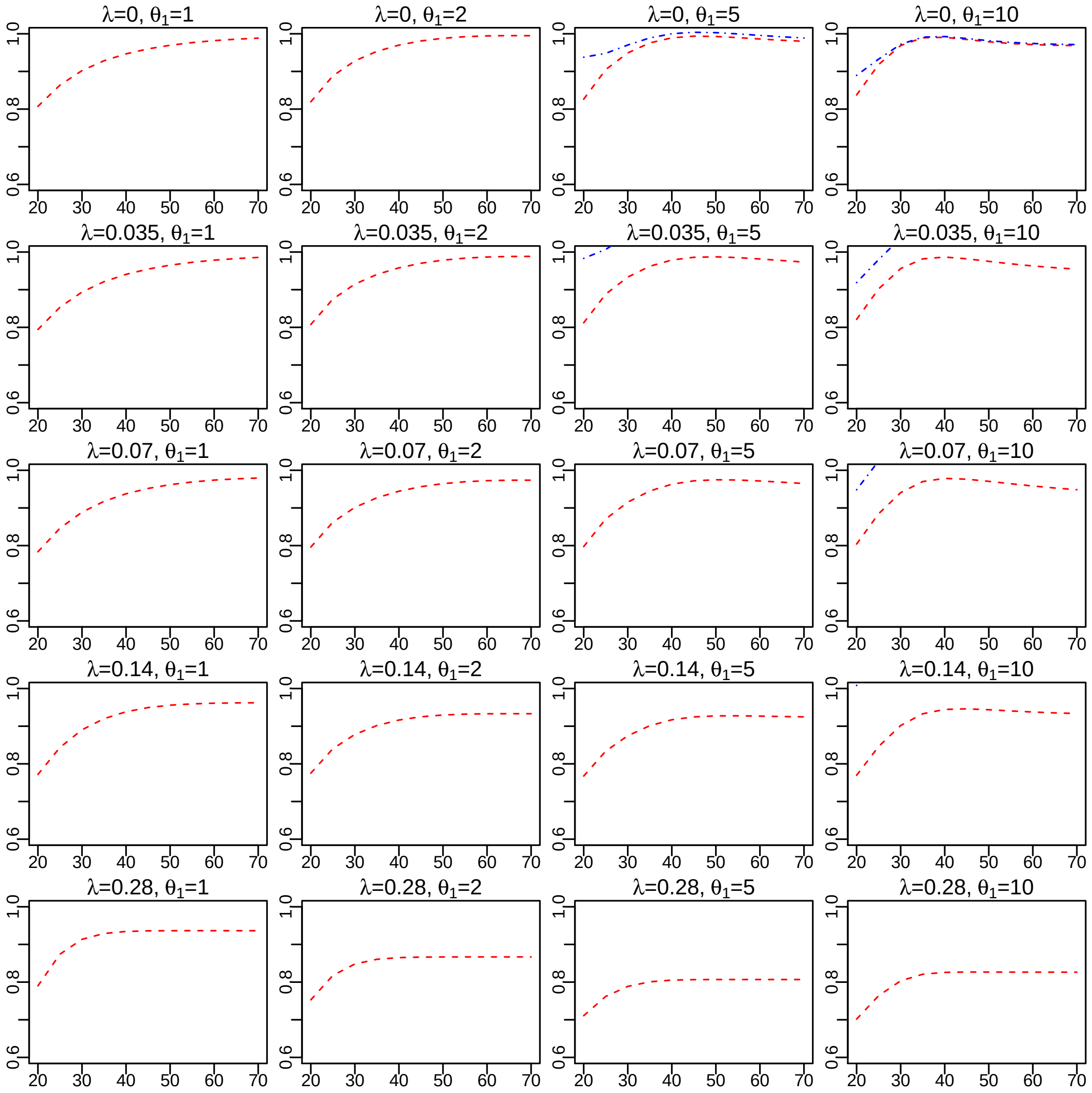

Figure A5: Asymptotic relative efficiency (ARE) of the estimator of $E(T)$ based on L_3 (red line) compared to L_1 with unknown λ. The x-axis of each graph is τ and the y-axis is the ARE. The final event times Y are double truncated within the interval $[10, \tau]$.

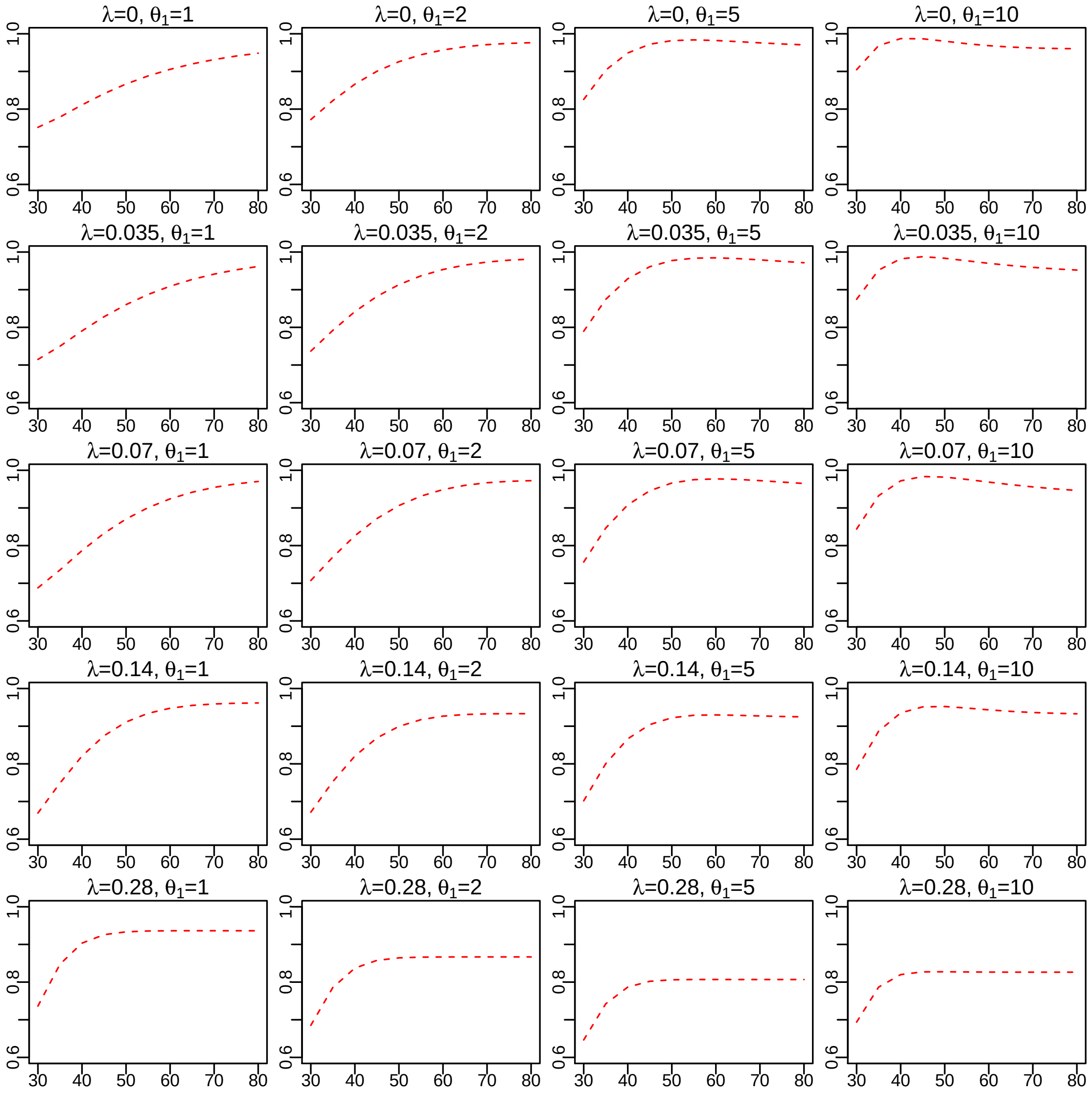

Figure A6: Asymptotic relative efficiency (ARE) of the estimator of $E(T)$ based on L_3 (red line) compared to L_1 with unknown λ. The x-axis of each graph is τ and the y-axis is the ARE. The final event times Y are double truncated within the interval $[20, \tau]$.

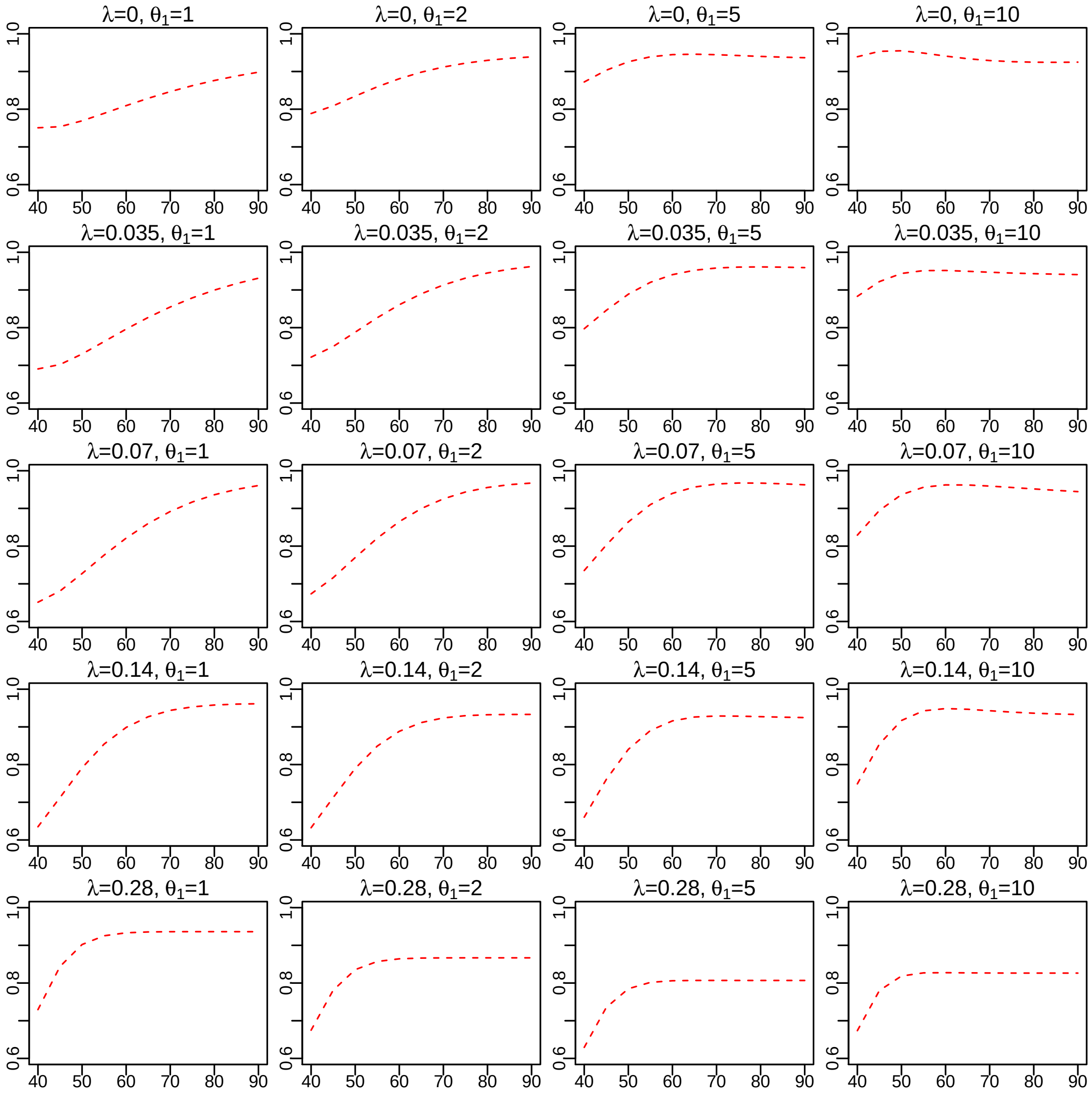

Figure A7: Asymptotic relative efficiency (ARE) of the estimator of $E(T)$ based on L_3 (red line) compared to L_1 with unknown λ. The x-axis of each graph is τ and the y-axis is the ARE. The final event times Y are double truncated within the interval $[30, \tau]$.

To compare more directly with Brookmeyer and Gail's result, we also calculated the ARE of L_3 with *known* θ_1 compared to L_1 with known λ *and known* θ_1. We found this ARE to be 0.20. Finally, we verified that when the right and left-truncation times were exactly 43.75 and 28.13, respectively, the ARE was 0.16, exactly the ARE that Brookmeyer and Gail calculated.

In the other scenarios considered by Brookmeyer and Gail, X was uniformly distributed over a shorter interval than $[0, \tau]$. They chose this distribution because they were motivated by the problem of estimating time from HIV infection to AIDS onset in haemophiliacs infected by receiving contaminated blood. The risk of infection by the blood transfusion route fell almost to zero in 1985, due to the introduction of testing of donor blood. Brookmeyer and Gail also considered scenarios where $f_X(x) \propto \lambda x$. We have not considered these scenarios here.

C Additional results from CoVID-19 analysis

C.1 Sex and age effect estimates from parametric models

Table A1 shows the estimated parameters and standard errors (SEs) for the gamma models, together with the p-values of sex and age, and the model deviance. Age is fitted as either: a) as a categorical variable with four levels (≥ 85, 75–84, 65–74 and 0–64); or b) as an ordinal variable ('age trend') that equals 0 for age ≥ 85, 1 for age 75–84, 2 for age 65–74, and 3 for age 0–64 and is assumed to have a linear effect. Table A2 shows the corresponding results for the log normal models.

C.2 Results from Brookmeyer and Liao's method

Table A3 shows the estimated parameters and standard errors (SEs) for the Brookmeyer-Liao models, together with the p-values of sex and age, and the model deviance.

C.3 Results from Vakulenko-Lagun et al.'s Cox regression method

Problems calculating the estimator

Vakulenko-Lagun et al.'s method uses inverse probability weighting to account for the right truncation, with the weights being functions of the Kaplan-Meier estimator, $\hat{G}(t)$, of the survivor curve for the truncation time variable calculated assuming left truncation by the delay. In order for this method to work for a particular data set, it has to be possible to calculate $\hat{G}(T_i)$ for all individuals i in the data set. This is, however, not guaranteed to be possible, as we now show.

Suppose, for ease of argument, that the truncation time is a discrete random

model	covariate	estimate	SE	p-value	deviance
1	shape	1.160	0.096		1558.6
	rate	0.059	0.018		
2	shape	1.195	0.099		1555.1
	rate	0.050	0.018		
	female	0.687	0.348	0.05	
3	shape	1.214	0.100		1550.8
	rate	0.103	0.026		
	age 75–84	−0.509	0.368	0.05	
	age 65–74	−1.332	1.022		
	age 0–64	−1.670	1.691		
4	shape	1.240	0.103		1549.0
	rate	0.090	0.027		
	female	0.430	0.302	0.15	
	age 75–84	−0.456	0.348	0.11	
	age 65–74	−1.115	0.802		
	age 0–64	−1.267	1.285		
5	shape	1.214	0.100		1550.8
	rate	0.104	0.025		
	age trend	−0.572	0.274	0.04	
6	shape	1.240	0.102		1549.0
	rate	0.090	0.027		
	female	0.428	0.299	0.15	
	age trend	−0.489	0.250	0.05	

Table A1: Parametric gamma models

model	covariate	estimate	SE	p-value	deviance
1	meanlog	3.442	0.570		1564.4
	sdlog	1.662	0.194		
2	meanlog	3.491	0.568		1543.8
	sdlog	1.639	0.189		
	female	−0.285	0.361	0.43	
3	meanlog	2.904	0.484		1538.2
	sdlog	1.594	0.177		
	age 75–84	0.497	0.386	0.10	
	age 65–74	0.841	0.602		
	age 0–64	1.515	0.788		
4	meanlog	2.968	0.500		1538.0
	sdlog	1.581	0.175		
	female	−0.192	0.324	0.55	
	age 75–84	0.473	0.382	0.12	
	age 65–74	0.830	0.593		
	age 0–64	1.453	0.780		
5	meanlog	2.904	0.475		1538.4
	sdlog	1.594	0.177		
	age trend	0.475	0.206	0.02	
6	meanlog	2.957	0.485		1538.0
	sdlog	1.580	0.175		
	female	−0.197	0.341	0.56	
	age trend	0.459	0.204	0.02	

Table A2: Parametric log normal models

model	covariate	estimate	SE	p-value	deviance
1	-	-	-	-	1534.8
2	female	−0.045	0.123	0.71	1534.6
3	age 75–84	0.142	0.137	0.21	1530.2
	age 65–74	0.181	0.209		
	age 0–64	0.474	0.223		
4	female	−0.036	0.123	0.77	1530.1
	age 75–84	0.142	0.147	0.21	
	age 65–74	0.179	0.210		
	age 0–64	0.472	0.223		
5	age trend	0.137	0.065	0.03	1530.5
6	female	−0.034	0.123	0.78	1530.4
	age trend	0.136	0.065	0.04	

Table A3: Brookmeyer and Liao's (1990) semi-parametric method

$P(T \geq \tau \mid \text{age} \geq 75)$	0.0	0.1	0.2	0.3	0.4	0.5	0.6	0.7	0.8	0.9
% failed	0.0	0.0	0.1	0.7	2.1	4.0	5.7	7.2	10.1	13.2

Table A4: Proportion of bootstrap samples for which the algorithm for fitting the Cox model for the effect of age on delay failed to converge

variable. In this case, $\hat{G}(t)$ is given by

$$\hat{G}(t) = \prod_{j=0}^{t-1} \left(1 - \frac{d_j}{r_j}\right) \qquad (t = 1, 2, \ldots \tau),$$

where d_j is the number of individuals in the sample whose truncation time $\tau - X$ equals j, and r_j is the number of individuals whose truncation time $\tau - X$ is greater than or equal to j and whose delay T is less than or equal to j. That is,

$$d_j = \sum_{i=1}^{n} I(\tau - X_i = j) \qquad \text{and} \qquad r_j = \sum_{i=1}^{n} I(T_i \leq j \leq \tau - X_i).$$

If $r_j = 0$ for some j (in which case d_j must also equal zero), then $\hat{G}(t)$ is undefined for all $t > j$ (because 0/0 is undefined).

In the CoVID data, there are two individuals who have delay T and truncation time $\tau - X$ both strictly greater than 44 days. All the other 302 individuals in the sample have T and $\tau - X$ both strictly less than 44. Thus, there are no individuals with truncation times of at least 44 and delays of at most 44, with the result that $r_{44} = 0$. Hence, $\hat{G}(t)$ is undefined for all $t \geq 45$. This is a problem, because two individuals have delays of at least 45 days.

To overcome this problem, we removed all individuals whose onset time was before 1st March. This had the effect of eliminating, in particular, the two problematic individuals from the sample. The Kaplan-Meier estimator $\hat{G}(t)$ could then be calculated using the remaining sample. Now $\hat{G}(T_i)$ was defined for all individuals in the (remaining) sample.

Results from the model including age

Figure A8 shows how the estimated log hazard ratio associated with age 0–74 (relative to age ≥ 75) changes as $P(T \geq \tau \mid Z = 0) = 1 - F_T^*(\tau \mid Z = 0)$ changes. Here $Z = 0$ means age ≥ 75. The proportion of bootstraps samples for which the fitting algorithm failed to converge are shown in Table A4.

References

[1] C Berg and HL Pedersen. The Chen-Rubin conjecture in a continuous setting. *Methods And Applications Of Analysis*, 13:63–88, 2006.

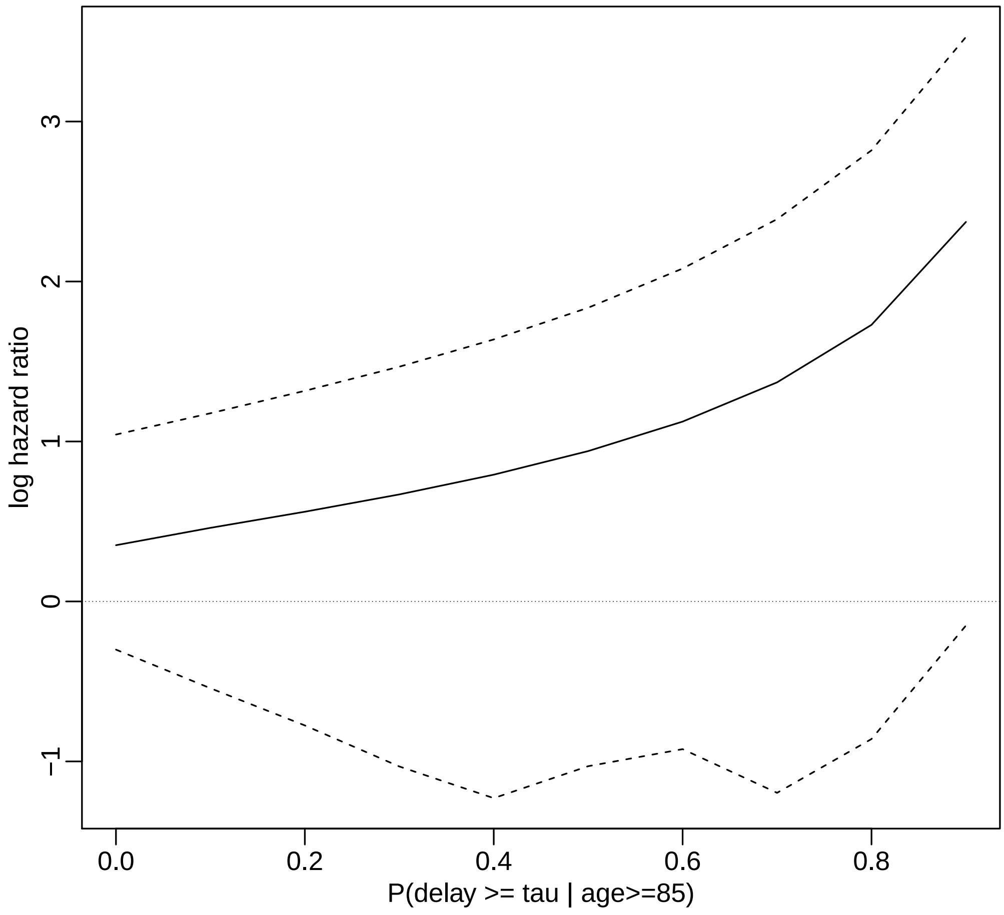

Figure A8: Estimate of log hazard ratio associated with age 0–74 as a function of $P(T \geq \tau \mid \text{age} \geq 75)$. Dotted lines indicate 95% confidence limits calculated by bootstrap; these may be unreliable when $P(T \geq \tau \mid \text{age} \geq 75)$ is large (see text).

[2] R Brookmeyer and MH Gail. A method for obtaining short-term projections and lower bounds on the size of the AIDS epidemic. *Journal of the American Statistical Association*, 83:301–308, 1988.
